# Compartmentalization proteomics revealed endolysosomal protein network changes in a goat model of atrial fibrillation

**DOI:** 10.1016/j.isci.2024.109609

**Published:** 2024-03-28

**Authors:** Thamali Ayagama, Philip D. Charles, Samuel J. Bose, Barry Boland, David A. Priestman, Daniel Aston, Georgina Berridge, Roman Fischer, Adam P. Cribbs, Qianqian Song, Gary R. Mirams, Kwabena Amponsah, Lisa Heather, Antony Galione, Neil Herring, Holger Kramer, Rebecca A. Capel, Frances M. Platt, Ulrich Schotten, Sander Verheule, Rebecca A.B. Burton

**Affiliations:** 1 Department of Pharmacology, University of Oxford, Oxford, UK; 2 Target Discovery Institute, University of Oxford, Oxford, UK; 3 Department of Pharmacology and Therapeutics, University College Cork, Cork, Ireland; 4 Department of Anaesthesia and Critical Care, Royal Papworth Hospital NHS Foundation Trust, Papworth Road, Cambridge CB2 0AY, UK; 5 Nuffield Department of Orthopaedics Rheumatology and Musculoskeletal Nuffield Department of Orthopaedics, Rheumatology and Musculoskeletal Sciences, Botnar Research Centre, University of Oxford, Headington OX3 7LD, UK; 6 Centre for Mathematical Medicine & Biology, Mathematical Sciences, University of Nottingham, Nottingham NG7 2RD, UK; 7 Department of Physiology, Anatomy and Genetics, , University of Oxford, South Park Road, Oxford OX1 3PT, UK; 8 Mass spectrometry Facility, The MRC Laboratory of Molecular Biology, Francis Crick Avenue, Cambridge CB2 0QH, UK; 9 Departments of Physiology and Cardiology, Cardiovascular Research Institute Maastricht, Maastricht University, Maastricht, the Netherlands; 10 University of Liverpool, Department of Pharmacology and Therapeutics, Institute of Systems, Molecular and Integrative Biology, Liverpool, UK

**Keywords:** Molecular biology, Cell biology, Proteomics, Transcriptomics

## Abstract

Endolysosomes (EL) are known for their role in regulating both intracellular trafficking and proteostasis. EL facilitate the elimination of damaged membranes, protein aggregates, membranous organelles and play an important role in calcium signaling. The specific role of EL in cardiac atrial fibrillation (AF) is not well understood. We isolated atrial EL organelles from AF goat biopsies and conducted a comprehensive integrated omics analysis to study the EL-specific proteins and pathways. We also performed electron tomography, protein and enzyme assays on these biopsies. Our results revealed the upregulation of the AMPK pathway and the expression of EL-specific proteins that were not found in whole tissue lysates, including GAA, DYNLRB1, CLTB, SIRT3, CCT2, and muscle-specific HSPB2. We also observed structural anomalies, such as autophagic-vacuole formation, irregularly shaped mitochondria, and glycogen deposition. Our results provide molecular information suggesting EL play a role in AF disease process over extended time frames.

## Highlights

• The EL proteins GAA, Rab7a, CLTB, VPS25, and CCT2 were significantly regulated
• Increased autophagic flux in AF goat model
• Glycogen accumulation and lysosomal-α-glucosidase upregulation
• Increased vesicular trafficking

## Graphical abstract

**Figure:**
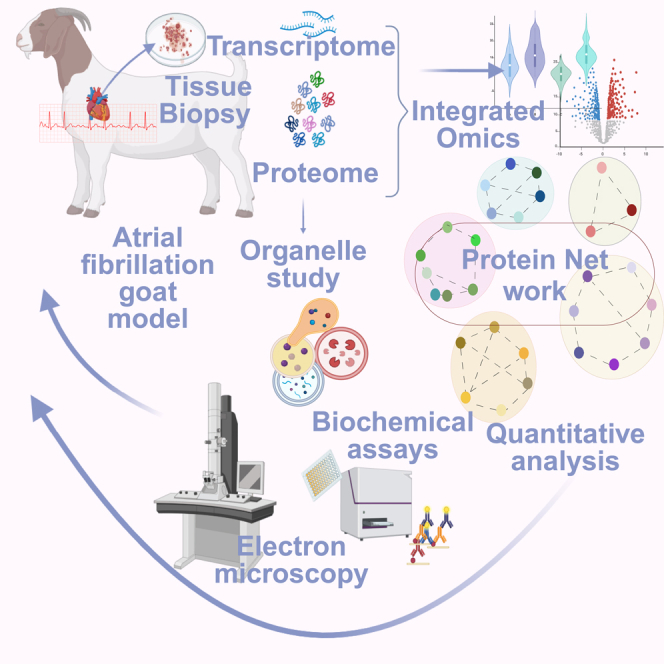

## Introduction

Atrial fibrillation (AF) is the most common sustained cardiac arrhythmia, accounting for around 14% of all strokes in the UK, and linked to a significantly high risk of developing embolic stroke.^1^^,^^2^ The prevalence of AF in the general population is 2%, although the risk of AF is age-dependent and this figure rises to 3.7–4.2% in ages 60–70 and 10–17% in the over 80s.^3^ AF is a progressive disease, progressing from paroxysmal to persistent forms, with self-sustaining progression driven by AF-triggered cardiac remodeling^4^^,^^5^ and potentially progression of comorbidities associated with AF.^6^ Atrial remodeling in AF can be the result of structural^7^^,^^8^ or electrical^9^ changes, and organelle dysfunction is also observed in AF progression.^10^

Regional and cell-type specific quantitative proteomics studies have enabled significant progress to be made in the understanding of the proteomic and transcriptomic contribution to AF pathology.^11^ However, the contribution of organelle remodeling in AF has not been studied extensively, including the potential contribution of changes in acidic organelles^12^ such as lysosomes and endolysosomes (EL), which play key roles in cellular energy metabolism^13^ and the trafficking of cellular components.^14^^,^^15^ Lysosomal changes have long been linked to cardiac disease,^16^ and changes in acidic organelles may be linked to underlying alterations in AF molecular pathways.^17^ Lysosomes may be linked to AF progression, for example via changes in autophagy.^18^ Indeed, several studies have recently identified autophagy as a potential mechanism underlying cardiac remodeling in AF progression,^18^^,^^19^ and autophagy has been shown to be increased in AF patients.^18^ However, little is known of how lysosomal proteins may be altered in AF. Techniques that enable proteomic characterization at the level of individual organelles,^20^^,^^21^ therefore, have the potential to highlight such changes. We have previously developed a modified density gradient method to isolate endolysosomal proteins, which increases the identification of endo-membrane proteins that are trafficked to acidic organelles,^21^ we applied this endolysosomal isolation method to conduct a proteomic study in a large animal AF model. We combined transcriptomics and proteomics to obtain the mRNA-protein correlation. In addition, we carried out high resolution electron microscopy imaging to confirm structural changes that have previously been reported in goat AF studies^22^^,^^23^ and we specifically look at acidic organelles at the cellular level.

The endolysosomal system consists of a series of membranous vesicles, composed of early endosomes, recycling endosomes, late endosomes and lysosomes. Autophagosomes are responsible for delivering the intracellular contents to lysosomes to complete autophagy. The endocytic pathway consists of acidification of the endosomes, maturation of endosomes to lysosomes accompanied by vesicle trafficking, protein sorting and targeted degradation of mostly sorted cargo. The two opposing sorting systems that are operating in these processes include the endosomal sorting complex required for transport (ESCRT, supports targeted degradation) and the retromer (supports retrograde retrieval of cargo). The EL system is emerging as a central player in a host of neurodegenerative diseases^24^ and its relevance in other diseases including AF is now being explored.

The present study was conducted using the AF goat as the animal model. The goat model is an ideal substitute for human AF as it has better tolerance than most contemporary animal models for AF, and the goat model is more comparable physiologically to humans compared to other models.^25^ The goat is a suitable model for conducting long-term cardiac pacing to develop sustained AF,^4^ and has been successfully utilized for studying electrical, contractile and structural remodeling in sustained AF pathology.^26^^,^^27^^,^^28^^,^^29^ During prolonged pacing, the AF goat model has been shown to undergo structural remodeling through endomysial fibrosis.^28^^,^^30^ Similar to humans, the AF goat model demonstrates the development of electrical conduction disturbances that give rise to complex activation patterns and endocardial-epicardial dissociation, providing a suitable substrate for the development of atrial arrhythmia. Studies from Wijffels et al.^31^ and Eijsbouts et al.^32^ highlight the relevance of the AF goat model’s suitability for antiarrhythmic drug targeted studies.

Our integrated approach combines transcriptomics and proteomics to provide a comprehensive comparative analysis of omics data that includes post-translational data. Dysregulated proteins were identified by performing label-free quantitative mass spectrometric analysis of the AF goat peptides compared to the sham goat models. After identifying dysregulated proteins, molecular pathways were used to understand how these dysregulations potentially affected the lysosomes and acidic organelles. Molecular pathways were analyzed using Cytoscape 3.7.2 with STRING, Kyoto Encyclopedia of Genes and Genomes (KeGG), Gene Ontology (GO), and Reactome pathway annotations to predict the relevant pathways. Furthermore, using GO, an over-representation study was performed. The most significantly regulated proteins identified in the AF goat model were compared against the human proteome, and the highest represented biological processes and cellular components that were altered in the AF goat model were identified based on comparison against human data.

## Results

### Identifying differential protein expression by quantitative proteomic analysis

**Figure 1:**
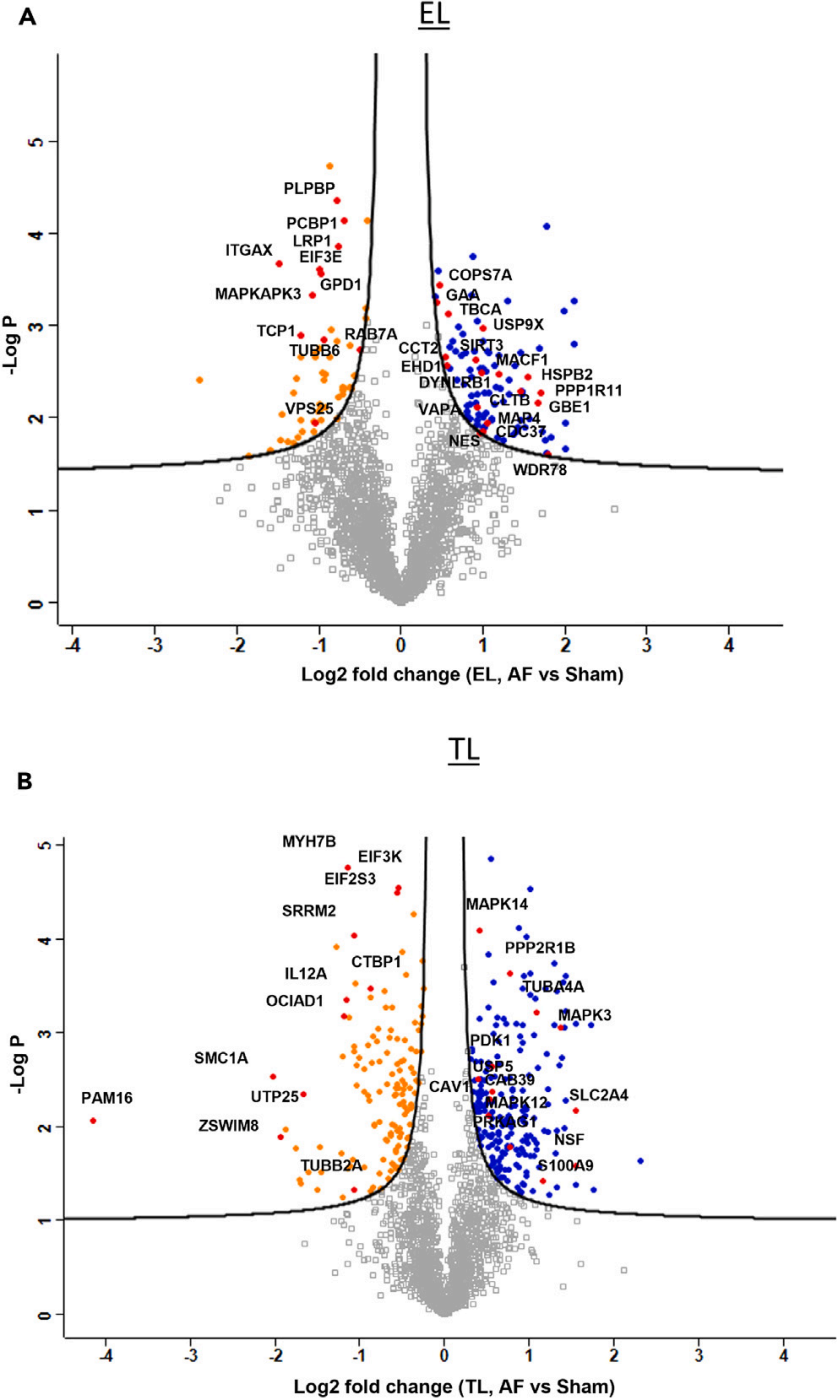
Volcano plot analysis (A and B) Volcano plot analysis of Endolysosome fraction (EL) and Tissue Lysate (TL) of the AF vs. sham goat models showing significantly upregulated (Blue) and downregulated (Orange) proteins in the AF (*p* = 0.05 and FDR = 0.05). Some of the most important proteins discussed are labeled in red. The –log2 transformed *p* values are plotted against the differentially regulated levels of Proteins in AF and sham.

A density gradient approach^21^ (see STAR methods) was used to isolate fractions corresponding to whole tissue lysate (TL), mitochondria (Mito) and endolysosomal lysate from sham (*N* = 3) and AF (*N* = 3) left atrial tissue biopsies from goat hearts. Tissue samples were then prepared as described in STAR methods for liquid chromatography-tandem mass spectrometry (LC-MS). The differential protein-expression levels between AF and sham groups were identified by quantitatively analyzing the mass spectrometric data of TL and EL using the Perseus software platform^33^ (version 1.6.15.0) (Figure 1A and 1B). The protein intensity values of each biological replicate were separated into protein groups (AF and sham) and imported into Perseus. These data matrixes were filtered by removing proteins with more than two missing values and used for quantitative analysis.

After the filtration, TL and EL samples each remained with 2104 proteins. Data were log-transformed (log2) and normalized via *Z* score. Data imputation was not required due to Progenesis reporting signal noise in absence of peptide precursors. Volcano plots were generated for each TL and EL sample by applying a two-way Student’s *t* test to identify the significant differences in protein regulations between AF and sham conditions (Figures 1A and 1B). The regulation levels were detected using a permutation-based false-discovery rate (FDR) of 5% with 250 randomizations at S0 = 0.1 and 99% confidence level.

**Figure 2:**
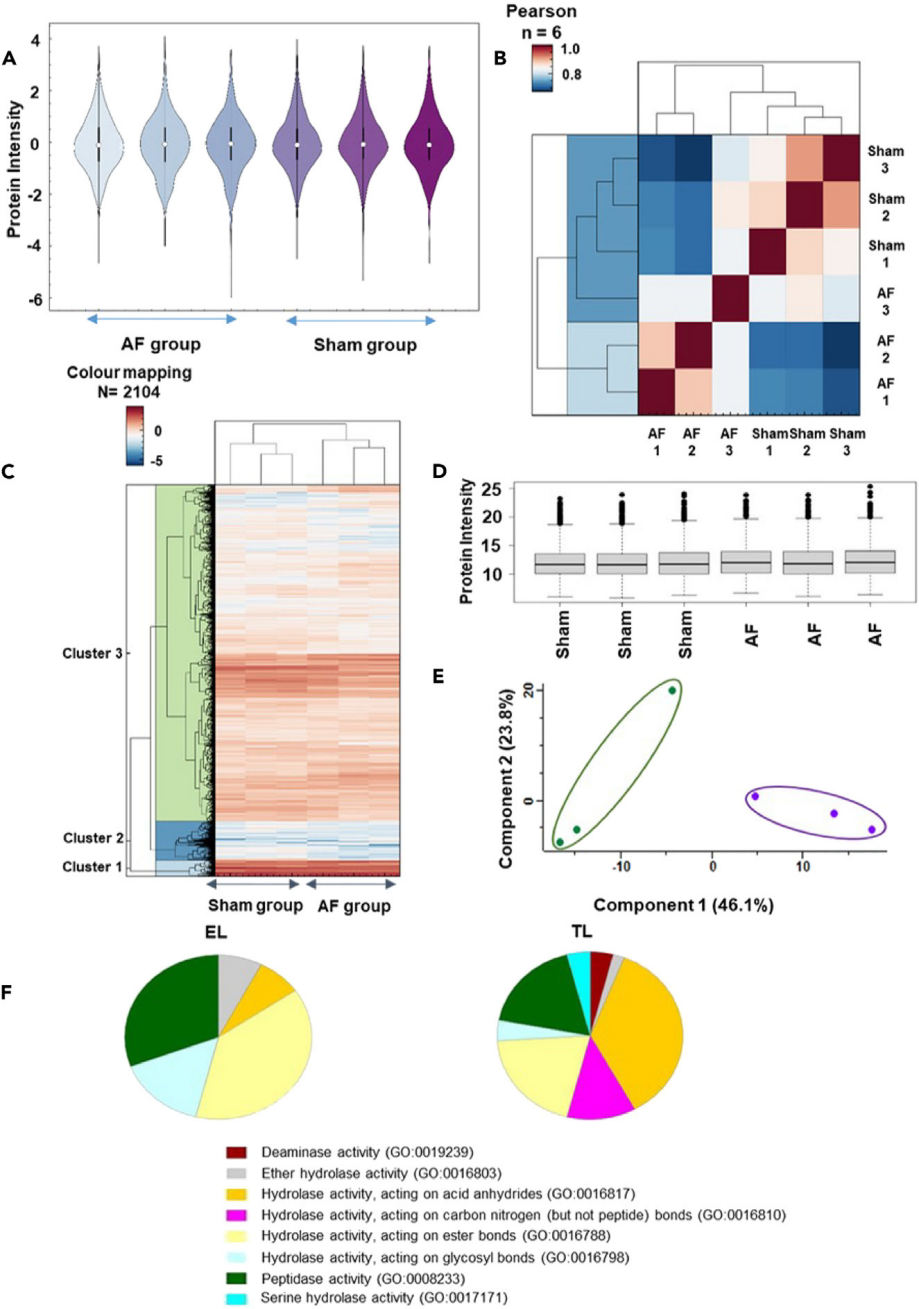
Proteomics data quantification (A) Violin plot shows protein intensity levels in the triplicated AF vs. sham EL samples. (B) Pearson co-efficient correlation plot explains the direct correlation between the sample protein intensities (1 = highest and 0 = lowest). (C) Heatmap for the AF and sham EL Samples demonstrating the z scored intensities of the differentially expressed proteins after unsupervised hierarchical clustering. (D) Whisker plot of the EL AF vs. sham protein intensity distribution shows the median, interquartile and the minimum to maximum outlier distribution. (E) Principal Component Analysis (PCA) demonstrating the spatial resolution among the averaged vectors of the AF vs. sham goat EL samples, and component one and two variations presents respectively 46.1% and 23.8%. (F) Gene ontology panther pathway analysis for EL and TL fractions. The molecular function of the endo-lysosome fraction (EL) showed 25% of catalytic activity, compared to the tissue lysate (TL) which showed 26.3% of catalytic activity. The catalytic hydrolase activity was further analyzed, and compared to TL (18%), EL fraction showed higher peptidase activity (30.8%), and hydrolases acting on ester bonds (TL = 20%, EL = 38.5%).

**Figure 3:**
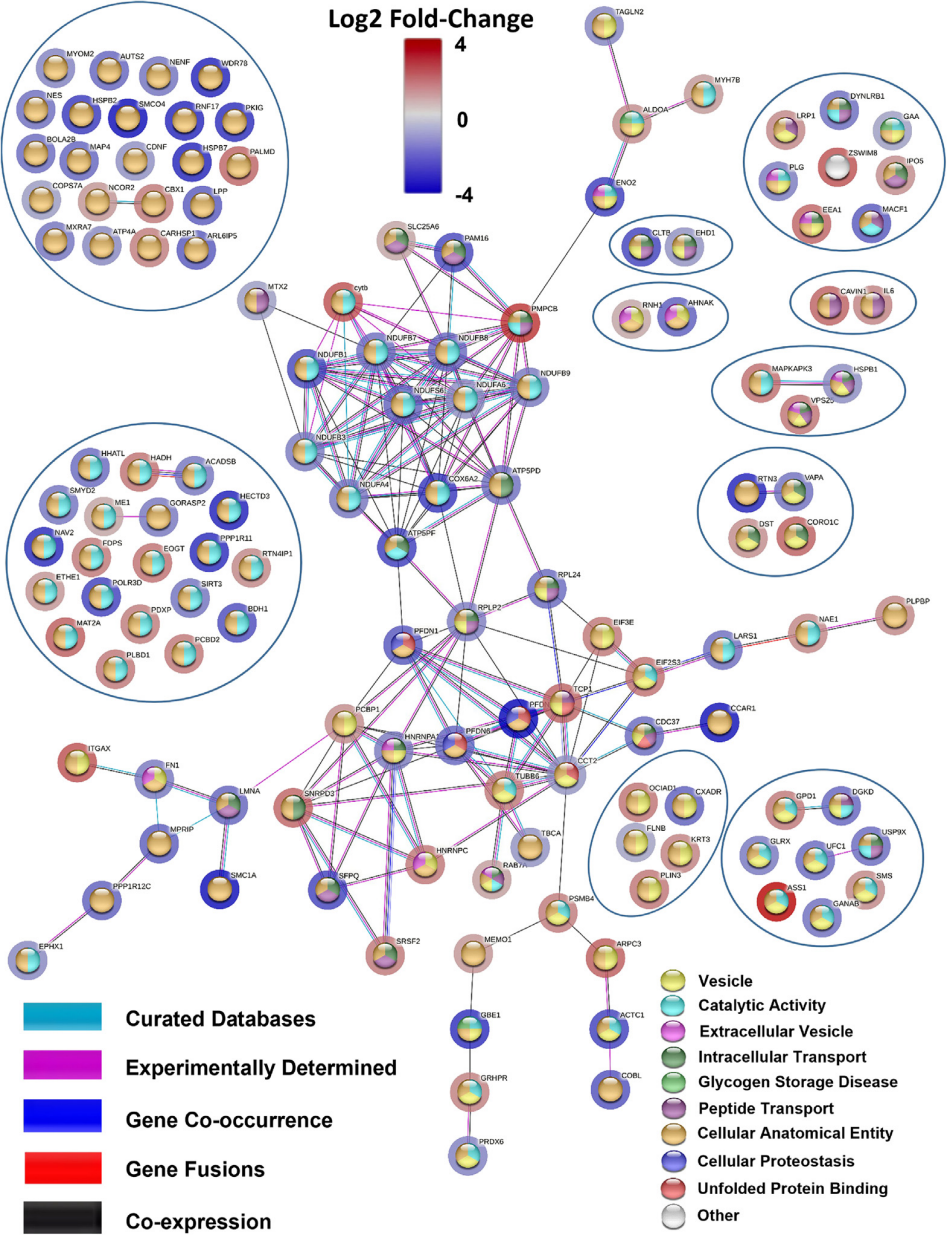
Using STRING network to study endolysosomal proteins Edges based on curated databases, experiments, gene co-occurrences, gene fusions, co-expressions and the nodes are colored according to their functional enrichments. The halos around the nodes display the significant log2 fold-change.

Violin plots (Figure 2A) were created using the kernel density estimation indicating the underlying distribution of the protein intensities between and within sham and AF group biological replicates by samples, color-coded with a gradient of purple, with higher protein intensity values presented as darker than the samples with comparatively lower intensities. Subsequently, Pearson coefficient correlation plots were created to observe the actual correlation between the groups (Figure 2B). Here the correlation ρ represents the interaction between two variables, or the AF and sham biological replicates, on a continuous scale of 1 to −1, where 1, depicts positive correlation, 0 depicts no correlation and −1 depicts negative correlation.^34^ Heat maps (Figure 2C) were created using Euclidian distance and K-mean clustering of the normalized protein intensities obtained from the quantified protein data matrix. A total of 2104 proteins in EL was observed in the three (EL) protein clusters. The red and blue color codes represent differential protein intensity levels and intensity whisker plots of each biological replicate were created to visualize the protein intensity distribution (Figure 2D). Principal Component Analysis (PCA) (Figure 2E) enabled observation of the vector distribution between and within the sample groups. In the PCA plots (Figure 2E), EL component 1 showed a 46.1% deviation respectively between AF (purple symbols) and sham (green symbols) groups. However, an exceptional segregation of 23.8% was observed in EL first biological replicates of sham groups, demonstrating that the variability between the differential experiment groups is more influential than the variability observed within the biological replicates. The most significantly regulated proteins of the EL fraction (Table 1) were further analyzed in STRING network to identify endolysosomal proteins with their respective structural or functional entities (Figure 3; Table 1).

**Table 1:** Contains the most significantly regulated endolysosomal (EL) fraction proteins of the AF goat model related to Figure 1A

Protein ID	Protein name	Gene	Fold change
A0A452F9W2_CAPHI	Prefoldin subunit 4	PFDN4	2.12
A0A452DN27_CAPHI	Single-pass membrane and coiled-coil domain-containing protein 4	SMCO4	2.01
A0A452DY79_CAPHI	Structural maintenance of chromosomes protein	SMC1A	2.01
A0A452DL17_CAPHI	SAP domain-containing protein	CCAR1	1.99
A0A452G2Z7_CAPHI	E3 ubiquitin-protein ligase HECTD3	HECTD3	1.83
A0A452DPX9_CAPHI	WD_REPEATS_REGION domain-containing protein	WDR78	1.79
A0A452EJX2_CAPHI	Reticulon	RTN3	1.78
A0A452G662_CAPHI	SHSP domain-containing protein	HSPB7	1.77
A0A452G8P6_CAPHI	RING finger protein 17	RNF17	1.75
A0A452DM61_CAPHI	Calponin-homology (CH) domain-containing protein	NAV2	1.72
A0A452F797_CAPHI	E3 ubiquitin-protein ligase PPP1R11	PPP1R11	1.71
A0A452FPZ5_CAPHI	cAMP-dependent protein kinase inhibitor	PKIG	1.68
A0A452G133_CAPHI	1,4-alpha-glucan branching enzyme	GBE1	1.67
A0A452F8B4_CAPHI	Cytochrome *c* oxidase subunit	COX6A2	1.56
A0A452G4I2_CAPHI	SHSP domain-containing protein	HSPB2	1.55
A0A452F5D8_CAPHI	2-phospho-D-glycerate hydro-lyase	ENO2	1.51
A0A452FLK7_CAPHI	ATP synthase-coupling factor 6, mitochondrial	ATP5PF	1.47
A0A452DQP7_CAPHI	Actin, alpha cardiac muscle 1	ACTC1	1.47
A0A452EBL3_CAPHI	DNA-directed RNA polymerase III subunit RPC4	POLR3D	1.46
A0A452F3N4_CAPHI	Clathrin light chain	CLTB	1.45
A0A452FRY3_CAPHI	Splicing factor, proline- and glutamine-rich	SFPQ	1.43
A0A452DRC3_CAPHI	Mitochondrial import inner membrane translocase subunit TIM16	PAM16	1.39
A0A452ERZ9_CAPHI	Complex I-MNLL	NDUFB1	1.37
A0A452G711_CAPHI	D-beta-hydroxybutyrate dehydrogenase, mitochondrial	BDH1	1.32
A0A452FQ01_CAPHI	Protein cordon-bleu	COBL	1.25
A0A452E6Y1_CAPHI	Prefoldin subunit 1	PFDN1	1.22
A0A452FJJ3_CAPHI	PRA1 family protein	ARL6IP5	1.2
A0A452DY71_CAPHI	Microtubule-actin cross-linking factor 1, isoforms 1/2/3/5	MACF1	1.19
A0A452FIK7_CAPHI	Lipoma-preferred partner	LPP	1.18
A0A452F973_CAPHI	Cathelicidin-2	CATHL2	1.16
A0A452EW34_CAPHI	Myosin phosphatase Rho-interacting protein	MPRIP	1.14
A0A452EQJ4_CAPHI	PDZ domain-containing protein	AHNAK	1.14
A0A452FP52_CAPHI	Coxsackievirus and adenovirus receptor	CXADR	1.11
A0A452F158_CAPHI	Protein phosphatase 1 regulatory subunit	PPP1R12C	1.1
A0A452FDC1_CAPHI	NADH dehydrogenase [ubiquinone] iron-sulfur protein 6, mitochondrial	NDUFS6	1.08
A0A452FQ33_CAPHI	Prelamin-A/C	LMNA	1.07
A0A452G3Z1_CAPHI	Diacylglycerol kinase	DGKD	1.06
A0A452EIY1_CAPHI	Microtubule-associated protein	MAP4	1.05
A0A452FHF8_CAPHI	Prefoldin subunit 6	PFDN6	1.04
A0A452EW73_CAPHI	Autism susceptibility gene 2 protein	AUTS2	1.04
A0A452EKC1_CAPHI	NADH dehydrogenase [ubiquinone] 1 beta subcomplex subunit 8, mitochondrial	NDUFB8	1.02
A0A452FL98_CAPHI	Ubiquitinyl hydrolase 1	USP9X	1
A0A452DMC5_CAPHI	Hsp90 chaperone protein kinase-targeting subunit	CDC37	1
A0A452FYK7_CAPHI	BolA-like protein 2	BOLA2B	1
A0A452E9A8_CAPHI	TRASH domain-containing protein	RPL24	1
A0A452FDZ6_CAPHI	Ubiquitin-fold modifier-conjugating enzyme 1	UFC1	1
A0A452FSE0_CAPHI	Protein-cysteine N-palmitoyltransferase HHAT-like protein	HHATL	1
A0A452F2G4_CAPHI	Dynein light chain roadblock	DYNLRB1	0.98
A0A452G757_CAPHI	IF rod domain-containing protein	NES	0.96
A0A452G5E3_CAPHI	ATP synthase subunit d, mitochondrial	ATP5PD	0.96
A0A452EGT0_CAPHI	Complex I-B18	NDUFB7	0.95
A0A452FP74_CAPHI	Gal_mutarotas_2 domain-containing protein	GANAB	0.94
A0A452ENN3_CAPHI	Leucyl-tRNA synthetase	LARS1	0.93
A0A452DKF1_CAPHI	Vesicle-associated membrane protein-associated protein A	VAPA	0.93
A0A452F4D3_CAPHI	Cystatin domain-containing protein	LOC102186806	0.92
A0A452EPW1_CAPHI	NAD-dependent protein deacetylase	SIRT3	0.91
A0A452G0B4_CAPHI	Cytochrome *b*5 heme-binding domain-containing protein	NENF	0.91
A0A452F760_CAPHI	N-lysine methyltransferase SMYD2	SMYD2	0.9
A0A452DZF9_CAPHI	Matrix-remodeling-associated protein 7	MXRA7	0.87
A0A452FYY0_CAPHI	Short/branched chain specific acyl-CoA dehydrogenase	ACADSB	0.87
A5JSS3_CAPHI	NADH dehydrogenase (Ubiquinone) 1 alpha subcomplex 4	NDUFA4	0.86
A0A452EKQ0_CAPHI	GRASP55_65 domain-containing protein	GORASP2	0.86
A0A452FF94_CAPHI	Glutaredoxin domain-containing protein	GLRX	0.85
A0A452FEX6_CAPHI	Complex I-B22	NDUFB9	0.85
A0A452FJK2_CAPHI	Fibronectin	FN1	0.84
PLMN_CAPHI	Plasminogen (Fragment)	PLG	0.81
A0A452FQM9_CAPHI	Complex I-B12	NDUFB3	0.81
A0A452G1G3_CAPHI	Helix-destabilizing protein	HNRNPA1	0.81
A0A452F1F7_CAPHI	60S acidic ribosomal protein P2	RPLP2	0.77
A0A452EUX2_CAPHI	Peroxiredoxin-6	PRDX6	0.76
A0A452E9Y7_CAPHI	Myomesin-2	MYOM2	0.74
A0A452GB99_CAPHI	Transgelin	TAGLN2	0.71
A0A452F2I2_CAPHI	Epoxide hydrolase	EPHX1	0.68
A0A452FAW7_CAPHI	Sodium/potassium-transporting ATPase subunit alpha	ATP4A	0.67
A0A452ESU7_CAPHI	Heat shock 27 kDa protein	HSPB1	0.63
A0A452F8A8_CAPHI	Cerebral dopamine neurotrophic factor	CDNF	0.6
A0A452EI29_CAPHI	NADH dehydrogenase [ubiquinone] 1 alpha subcomplex subunit 6	NDUFA6	0.6
A0A452FI28_CAPHI	Tubulin-specific chaperone A	TBCA	0.58
A0A452FFI1_CAPHI	EH domain-containing protein 1	EHD1	0.57
A0A452G4D6_CAPHI	CCT-beta	CCT2	0.55
A0A452EF68_CAPHI	PCI domain-containing protein	COPS7A	0.48
A0A452DXZ3_CAPHI	Metaxin-2	MTX2	0.46
A0A452EV92_CAPHI	P-type domain-containing protein	GAA	0.44
A0A452ETD8_CAPHI	Filamin-B	FLNB	0.41
A0A452ERC8_CAPHI	Ribonuclease inhibitor	RNH1	−0.41
A0A452EB34_CAPHI	ADP/ATP translocase 3	SLC25A6	−0.43
A0A452FQE3_CAPHI	Malic enzyme	ME1	−0.43
A0A452DMR1_CAPHI	Ras-related protein Rab-7a	RAB7A	−0.5
A0A452F3X0_CAPHI	Myosin-7B	MYH7B	−0.57
A0A452DSU6_CAPHI	Mediator of ErbB2-driven cell motility 1	MEMO1	−0.61
A0A452EI23_CAPHI	Lactamase_B domain-containing protein	ETHE1	−0.62
A0A452E1G5_CAPHI	Nuclear receptor corepressor 2	NCOR2	−0.62
A0A452DVL8_CAPHI	Poly(rC)-binding protein 1	PCBP1	−0.7
A0A452G6A1_CAPHI	Dystonin	DST	−0.7
A0A452F3M1_CAPHI	Importin N-terminal domain-containing protein	IPO5	−0.7
A0A452EH63_CAPHI	Fructose-bisphosphate aldolase	ALDOA	−0.72
A0A452DZ25_CAPHI	NEDD8-activating enzyme E1 regulatory subunit	NAE1	−0.76
A0A452FYK1_CAPHI	Exosome complex protein LRP1	LRP1	−0.76
A0A452FU89_CAPHI	Pyridoxal phosphate homeostasis protein	PLPBP	−0.78
A0A452DU00_CAPHI	PABS domain-containing protein	SMS	−0.79
A0A452E7Y7_CAPHI	PKS_ER domain-containing protein	RTN4IP1	−0.79
IL6_CAPHI	Interleukin-6	IL6	−0.86
A0A452FN18_CAPHI	IF rod domain-containing protein	KRT3	−0.86
A0A452FAD3_CAPHI	Glyoxylate reductase/hydroxypyruvate reductase	GRHPR	−0.87
A0A452EQN7_CAPHI	CSD_1 domain-containing protein	CARHSP1	−0.92
A0A452EKB8_CAPHI	Pyridoxal phosphate phosphatase	PDXP	−0.92
A0A452FAZ1_CAPHI	Proteasome subunit beta	PSMB4	−0.94
A0A452G1A9_CAPHI	Tubulin beta chain	TUBB6	−0.95
A0A452EXY9_CAPHI	Serine/arginine-rich splicing factor 2	SRSF2	−0.95
A0A452FD14_CAPHI	Glycerol-3-phosphate dehydrogenase [NAD(+)]	GPD1	−0.97
A0A452FCU2_CAPHI	Farnesyl pyrophosphate synthase	FDPS	−0.98
A0A452E698_CAPHI	Chromobox protein homolog 1	CBX1	−0.98
A0A452FV62_CAPHI	4a-hydroxytetrahydrobiopterin dehydratase	PCBD2	−0.98
A0A452DRD5_CAPHI	Eukaryotic translation initiation factor 3 subunit E	EIF3E	−0.99
A0A452FVJ7_CAPHI	Phospholipase B-like	PLBD1	−0.99
A0A452FUU5_CAPHI	Perilipin	PLIN3	−1
A0A452FP83_CAPHI	RRM domain-containing protein	HNRNPC	−1
A0A452ES90_CAPHI	ESCRT-II complex subunit VPS25	VPS25	−1.04
A0A452FY19_CAPHI	OCIA domain-containing protein	OCIAD1	−1.04
A0A452EHJ6_CAPHI	Hydroxyacyl-coenzyme A dehydrogenase, mitochondrial	HADH	−1.05
A0A452EJR1_CAPHI	EGF domain-specific O-linked N-acetylglucosamine transferase	EOGT	−1.06
A0A452DQ55_CAPHI	Protein-synthesizing GTPase	EIF2S3	−1.07
A0A452EIV8_CAPHI	Non-specific serine/threonine protein kinase	MAPKAPK3	−1.08
A0A452DKT5_CAPHI	Small nuclear ribonucleoprotein Sm D3	SNRPD3	−1.21
A0A452FIX8_CAPHI	Early endosome antigen 1	EEA1	−1.21
A0A452EVX4_CAPHI	CCT-alpha	TCP1	−1.23
A0A452G5M5_CAPHI	Palmdelphin	PALMD	−1.23
A0A452FLJ3_CAPHI	Caveolae-associated protein 1	CAVIN1	−1.26
A0A452FNA0_CAPHI	Coronin	CORO1C	−1.28
A0A452G7L4_CAPHI	S-adenosylmethionine synthase	MAT2A	−1.31
A0A452FVB0_CAPHI	Actin-related protein 2/3 complex subunit 3	ARPC3	−1.32
A0A452FX49_CAPHI	Hemoglobin subunit beta-C	HBBC	−1.38
Q8WF85_CAPHI	Cytochrome *b*	cytb	−1.46
A0A452DJR4_CAPHI	SWIM-type domain-containing protein	ZSWIM8	−1.48
A0A452DS40_CAPHI	VWFA domain-containing protein	ITGAX	−1.48
A0A452F341_CAPHI	Beta-MPP	PMPCB	−1.86
A0A452F1Z3_CAPHI	Argininosuccinate synthase	ASS1	−2.45

### Confirmation of selected proteins by western blotting

**Figure 4:**
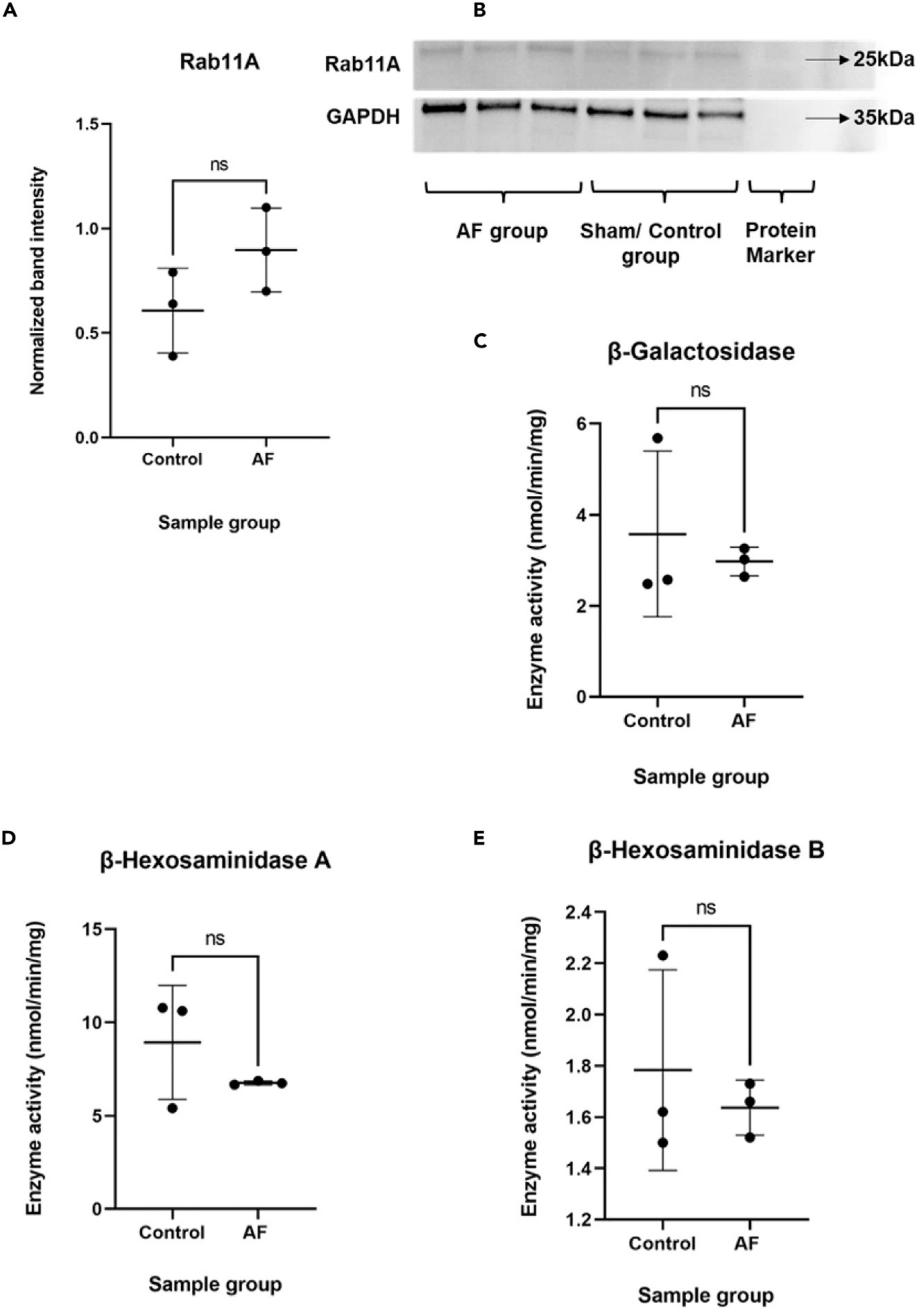
Protein identification using western blotting and lysosomal enzymatic Assays (A) Western blots were performed on *N* = 3 biological samples of each sham and AF group. Rab11A (24–25 kDa) and GAPDH control (37 kDa) were detected. (B) The normalized band intensities of Rab11A data are presented as mean ± SD. The normalized band intensity value for the control group was 0.61 ± 0.20, and for the AF group at 0.90 ± 0.20. After performing a one-way t-test, the normalized protein band intensity showed a trend toward a significant upregulation in the AF group (*p* = 0.07). (C) β galactosidase activity is displayed as mean ± SD. The AF group showed an enzymatic activity of 2.64, 3.02 and 3.26 nmol/min/mg, respectively, and the sham group showed 2.48, 2.57 and 5.68 nmol/min/mg. The mean enzymatic activity was 2.57 ± 0.31 for the AF group and 3.57 ± 1.8 for the control group. After performing a Two-way t-test, the β galactosidase activity of the AF group showed no significant regulation (*p* = 0.60). (D) β hexosaminidase type-A activity of the AF group (*N* = 3) showed 6.66, 6.74 and 6.87 nmol/min/mg, respectively, and the sham group showed 5.4, 10.61 and 10.78 nmol/min/mg activity. The normalized mean enzymatic activity was at 8.93 ± 3.1 for the control group and 6.75 ± 0.11 for the AF group, and β hexosaminidase type- A activity of the AF group showed no significant regulation (*p* = 0.28). (E) AF group showed (*N* = 3) 1.52, 1.66 and 1.73 nmol/min/mg of β hexosaminidase type B activity, and the sham group (*N* = 3) showed 1.5, 1.62 and 2.23 nmol/min/mg of β hexosaminidase type B activity, The mean enzymatic activity was at 1.8 ± 0.39 for the control group and 1.6 ± 0.11 for AF group. No significant regulation was observed in β hexosaminidase type B activity (*p* = 0.56).

The Ras-related protein Rab-11A (Rab11A) is expressed ubiquitously^35^^,^^36^ and plays important roles in intracellular transport. Rab11A was previously found to be significantly upregulated in the AF goat proteomics model. Western blotting was conducted on the same (*N* = 3) biological samples retrieved from sham and AF goat models to evaluate this previous finding (Figures 4A and 4B). GAPDH was used as the control protein, and intensities of Rab11A bands were normalized using the GAPDH band intensity. The normalized data are presented as mean ± SD. The normalized band intensity for Rab11A was at 0.61 ± 0.20 for the control group and 0.90 ± 0.20 for the AF group. The Rab11A upregulation in AF was analyzed by conducting a one-way t-test to analyze for upregulation. Although our data did not support a significant upregulation in Rab11A in the AF group, the *p*-value of 0.07 suggests a trend toward Rab11A upregulation in the AF group when compared to sham controls (*N* = 3) (Figures 4A and 4B). This difference between our data and data published previously by Lapierre et al.^35^ is likely the result of the lower power (*N* = 3) of our data.

### Lysosome hydrolase activity assays

To check for impairment of autophagy, we conducted biochemical assays to investigate whether lysosomal enzyme activity is changed in goat AF. Lysosomal enzyme activity assays were performed on the three most common lysosomal enzymes: β galactosidase, β-hexosaminidase type A, and β hexosaminidase type B (Figures 4C–4E). The enzymatic activities were analyzed using a two-way t-test and no significant difference was detected in any of the 3 lysosomal enzymes studied.

Altered lysosomal enzyme activities are observed in lysosomal storage disorders (LSDs),^37^ and some patients with LSDs show severe cardiac phenotypes.^38^ However, we did not observe such significant changes in the AF goat model, indicating little or no impairment in autophagy and this result is reinforced by the western blotting data on LC3I proteins (Figure S2A).

### mRNA transcript analysis using next generation sequencing

#### Distance matrix heatmap

The distances between samples were analyzed using hierarchical clustering to provide an overview of the similarities and dissimilarities between the samples.

Sample distances were displayed using a range of shaded color bar from dark blue to white, 0 being highly similar and displayed as dark blue and 200 being highly dissimilar represented in white color (Figure S3A).

#### Heatmap

The topmost regulated genes were plotted using hierarchical clustering to determine whether the samples cluster together according to the AF or sham conditions. All the AF and sham goat samples of LA tissue were clustered together, (Figure S3B).

#### PCA

PCA was performed to analyze the sample distribution and to assess the reliable correlation between the sample triplicates between AF and sham model groups by reduced data dimension for a simpler interpretation. As indicated in Figure S3C, vector deviation of 67.3% was observed at component 1 (PC1) between AF (purple symbols) and sham/control groups (green symbols). An exceptional 11.8% segregation was observed in component 2 (PC2), or the biological replicates within the groups. Since, PC1 value was higher than PC2, it can be concluded that the variance between the two conditions is higher than the variance within a group (Figure S3C).

#### Whisker plot

The transcript intensity distribution between AF and sham goat model samples were observed using whisker plot (Figure S3D).

The whole tissue lysates (same samples as used in proteomics and Western blotting) were used to isolate mRNA, genomic expression was quantified, and the differential expression plotted as a volcano plot (Figure S4A). These differentially expressed genes were fed into Reactome pathway analysis to study the pathway regulations. We observed a significant upregulation of 235 genes, while 297 genes were significantly down regulated (Table S1.1).

### Integrated analysis of transcriptomics and proteomics

The integrated omics analysis highlighted several regulated pathways that were categorized under three confidence levels. These are color coded in gray, black and red which represents non-confident, confident and highly confident pathways, respectively in Figures S4B and S5B. In our analysis and discussion, we do not consider non-confident pathways. Furthermore, the significance of these pathways was determined with a pathway score (PS) to show their enrichment levels in both transcriptomics and proteomics analysis.

Interestingly, our integrated analysis highlighted RHOBTB GTPase, RHOBTB1 and RHOBTB2 GTPase cycles to be significantly enriched in transcriptomics. While the integrated analysis did not highlight a significant enrichment in proteomics. The pathways, mitochondrial ABC transporters, interleukin (IL) 9 signaling, defective POMGNT1 causes MDDGA3, MDDGB3 and MDDGC3, beta-oxidation of pristanoyl-CoA, RUNX3 regulates RUNX1-mediated transcription, RUNX3 Regulates Immune Response and Cell Migration, arachidonate production from DAG, RUNX1 regulates estrogen receptor mediated transcription, abacavir metabolism, coenzyme A biosynthesis, suppression of autophagy, regulation of RUNX1 expression and activity, defective HK1 causes hexokinase deficiency (HK deficiency), activation of BMF and translocation to mitochondria, neurofascin interactions, degradation of GABA, and formation of the active co-factor, UDP-glucuronate are significantly downregulated in proteomics compared to transcriptomics. Some of the downregulated pathways in both the integrated TL proteomics and transcriptomics were, defective PMM2 causes PMM2-CDG (CDG-1a), defective MMAA causes methylmalonic aciduria type cblA, defective MUT causes methyl malonic aciduria mut type, glycogen storage disease types II, IV, XV and 0, defective PGM1 cause of PGM1-CDGII, MET activation of PI3K/AKT signaling, MET activation of PTPNII, inhibition of NO production, and stimulation of the cell death response by PAK-2P34 (labeled in red circles, Figure S4B and Table S2.1).

Like the TL fraction, the integrated analysis of the most significantly up and down regulated EL proteins and genes of transcriptomics analysis (Figure S5B) showed RHOBTB and RHOBTB2 GTPase cycles up regulated. From the integrated analysis, the pathways such as, signaling by PDGFRA transmembrane, juxtamembrane, and kinase domain mutants, signaling by cytosolic FGFR1 fusion mutants, ROBO receptors bind AKAP5, interconversion of 2-oxoglutarate and 2-hydroxyglutarate, IL-6 signaling, defective TPR may confer susceptibility toward thyroid papillary carcinoma (TPC), regulation of Glucokinase by Glucokinase Regulatory Protein, beta-oxidation of pristanoyl-CoA, defective MPI causes MPI-CDG (CDG-1b), suppression of autophagy, MAPK1 (ERK2) activation, phosphor-PLA2 pathway, degradation of GABA, IL-9 signaling, inhibition of NO production, formation of the active cofactor, UDP-glucuronate, and signaling by PDGFRA extracellular domain mutants were downregulated in proteomics compared to transcriptomics. Activation of PPARGC1A (PGC-1alpha) by phosphorylation, defective GFPT1 causes CMSTA1, stimulation of the cell death response by PAK-2P34, MET activation of PI3K/AKT signaling, glycogen storage disorders such as type II, IV, XV, and 0, non-canonical activation of NOTCH3, defective PGM1 cause of PGM1-CDGII, neurofascin interactions, LRR FLII-interacting protein 1 (LRRFIP1) activates type I IFN production, fibronectin matrix formation, mitochondrial ABC transporters, regulation of RUNX1 expression and activity, Tie2 signaling, defective MMAA causes methyl malonic aciduria type cblA, defective MUT causes methyl malonic aciduria mut type and MET activates PTPN11 were the significantly down regulated pathways in, both proteomics and transcriptomics. The regulation of GAP junction activity pathway was downregulated in TL proteomics compared to transcriptomics, (Figure S4B, and Table S2.1).

To identify and understand the overall representation of the pathways related to the EL, acidic organelles, and vesicle trafficking, the enrichment scores from the listed significant pathways of integrated proteomics and transcriptomics analysis were separated (Figure S5A and Table S2.1).

Furthermore, the integrated analysis of the most significantly up or downregulated EL, TL proteins and genes highlighted by the proteomic and transcriptomic analysis showed pathways related to the following as being confidently upregulated (black in color circled; Figures S4B and S5B and Table S2.1); membrane trafficking, vesicle mediated transport, TCA cycle and respiratory pathway, chaperone mediated protein folding, ER to Golgi anterograde transport, ER- Phagosome pathway, lipid metabolism, COPI mediated anterograde transport, while anabolic pathways such as rRNA processing, and cell cycle were among the down regulated pathways with confidence (the most significant list of pathways are summarized in Figures S4B and S5B and the complete list of the pathways are shown in the Table S2.1).

### Structural insights using electron microscopy (EM)

**Figure 5:**
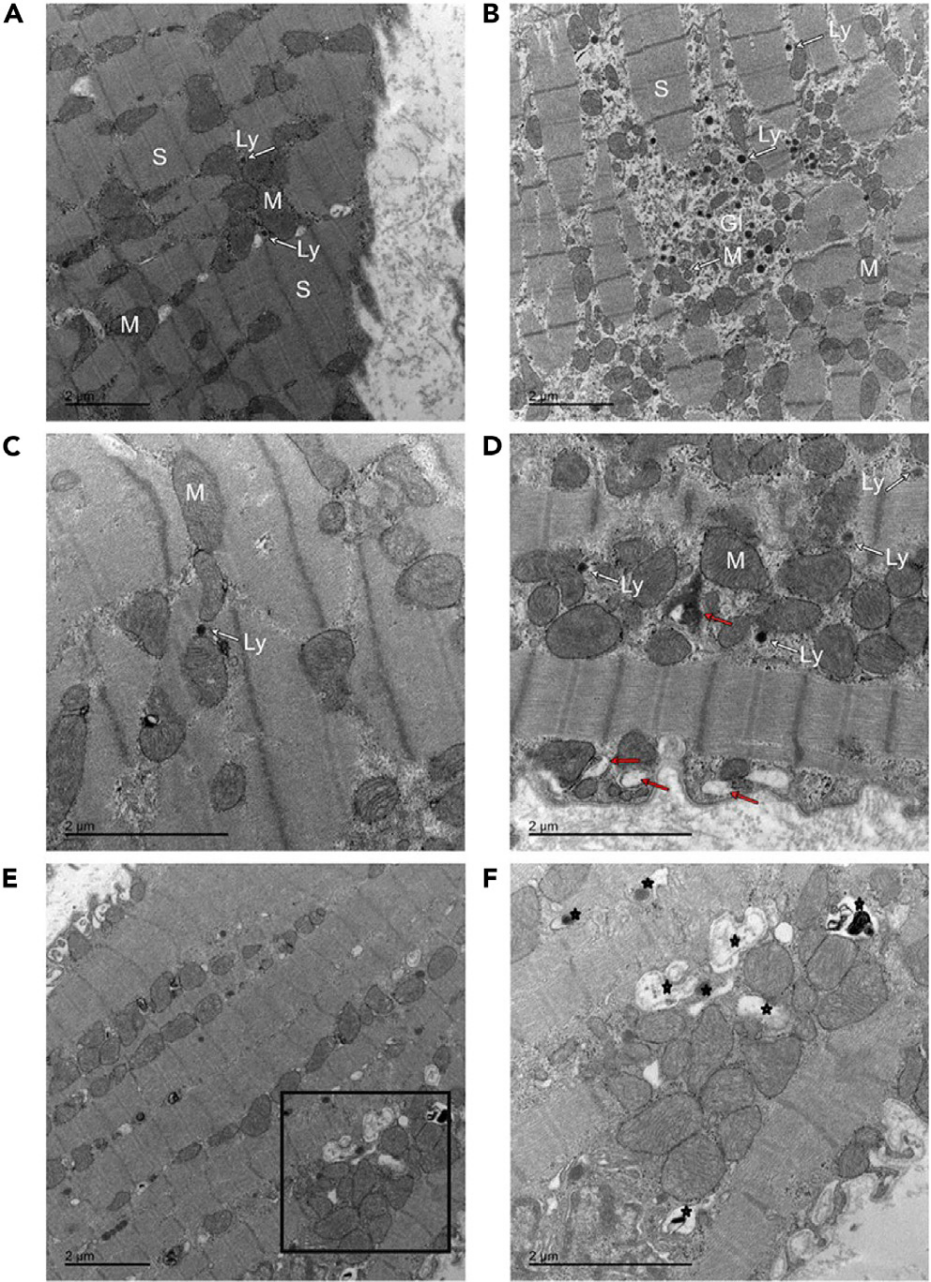
Electron microscopy images of goat left atrial myocardium tissue to identify structural changes Qualitative electron microscopy (EM) on goat left atrial myocardium tissue samples in sinus rhythm (sham, A and C) and after prolonged sustained atrial fibrillation (B, D, E and F). Panel A shows sarcomeres (S) regularly spaced and surrounded by mitochondria. Glycogen (GL), lysosomes (Ly) and numerous irregular shaped mitochondria observed in atrial fibrillation sample (B). We observe many irregular shaped mitochondria dispersed in myolytic areas, autophagic vacuoles (red arrows) and lysosomes (Ly) in (D). Electron micrographs highlighting increased number of endocytic vesicles and vacuoles including endosomes, autophagosomes, lysosomes in atrial fibrillation samples (E and F), indicated by star symbols). (F) is a higher resolution image of the marked square area in Panel E. Samples for EM were collected from *N* = 3 for AF and *N* = 3 for Sinus rhythm control animals.

We qualitatively analyzed EM images from goat AF and sham control tissue and observed that the sarcomeres were regularly distributed throughout the cytoplasm and there were rows of uniformly sized mitochondria between them in control samples (Figure 5A and 5C panels); similar to observations reported in.^39^ In AF tissue, we observed increased myolysis (Figure 5B and 5D), with areas depleted in sarcomeres and smaller, irregular mitochondria observed (Figure 5D). EL and autophagic vacuoles and autophagic-lysosomes are more commonly seen in AF tissue (Figure 5E and more zoomed in detail in Figure 5F). Since glycogen is associated with lysosomes or lysosome-like organelles,^40^ we quantitatively analyzed the amount of glycogen accumulation in our AF and sham tissue samples (Figure S6A). Both manual and automated counting observed increased glycogen levels in AF samples (manual disease mean glycogen count 6354 ± 714.8/nm^2^ vs. control mean glycogen count 3195 ± 417.9/nm^2^, *p* = 0.0023; Automated count disease mean glycogen count 6529 ± 555.0/nm^2^ vs. control mean glycogen count 4154 ± 415.8/nm^2^, *p* = 0.0003. *N* = 3 for each group. Figure S6B).

### The overrepresentation of *Capra hircus* (goat) protein regulation enrichment comparison to total human proteome and to the *Cavia porcellus* (guinea pig)

The significantly up and down regulated protein/gene names with the respective log2 fold-change values were then uploaded to the Panther–Gene Ontology (GO), and an overrepresentation test was conducted against the reference *H. sapiens* proteome (Taxonomy ID: 9606) (Figures S1A and S1B). This overrepresentation test was conducted on GO terms, Cellular Component (GOCC) and Biological Process (GOBP). The highest enriched GOCC term included muscle filaments, myofibrils proteasome complex, sarcoplasmic reticulum lumen, endocytic vesicles, and vesicle lumen (Figure S1A). In contrast, the highest enriched GOBP term included the energy metabolism and vesicle-mediated trafficking pathways (Figure S1B).

We performed a Venn analysis to understand the total protein yield of C. *hircus* TL and EL compared to the C. *porcellus* protein list that was published previously using this density-gradient based method from Ayagama et al.^21^ (Figure S1C). These comparisons showed a good overlap between goat and guinea pig data from these separate investigations, with 44.2% shared proteins for TL, and 28.8% shared proteins for EL samples. Due to the higher availability of annotation data for *H. sapiens*, the UniProt KB identifiers of the most significantly regulated *C. hircus* proteins identified from the TL volcano plot of the AF compared to the sham samples (Figure 1B) were converted to the *H. sapiens* identifiers.^21^

## Discussion

Previous studies investigating mechanisms of AF using the goat model have shown structural, electrical, contractile and molecular changes compared with sinus rhythm controls (some examples include Ausma et al.,^39^ Wijffels et al.^4^ Allessie et al.,^41^ van Hunnik et al.,^42^ and Neuberger et al.^43^). Proteomics studies have been performed in many cardiac diseases including AF^44^ and reviewed by^45^ and have highlighted the need for more multi-omics research to investigate possible implicated molecular pathways in the development of AF. In this study we have focused on changes in EL-related proteins as another factor with a slower time course of development, and their involvement in this disease. Our endolysosomal organelle proteomics approach contributes new insight into regulation and differential functionalities related to these pathways and molecular mechanisms observed in this large animal model of AF. Here, we provide data related to pathway dysregulations in endolysosomal proteins previously not explored. Analysis of such proteins in separated tissue lysate (TL) and endolysosome fractions (EL) increased our ability to uncover EL-specific proteins that were not identified in the TL, such as GAA, CLTB, DYNLRB1, SIRT3, CCT2, and muscle specific HSPB2^46^ (See Table S3.1 for the list of proteins). Combining an integrative multi-omics approach in this study helps us highlight interrelationships of the biomolecules and their functions in this disease and deriving insights into the data we have collected.

Lysosome number and dysfunction has been characterized in several cardiac conditions,^47^ including congenital atrial septal defects, and AF is a common complication in these patients.^48^ Further, degenerative changes, including accumulation of lysosomes, have been found to correlate with atrial cellular electrophysiological changes.^49^ Whilst AF is a common complication in atrial septal defect patients, no published studies have investigated the role of the EL or their corresponding interactive effects on other organelles in AF itself.

**Figure 6:**
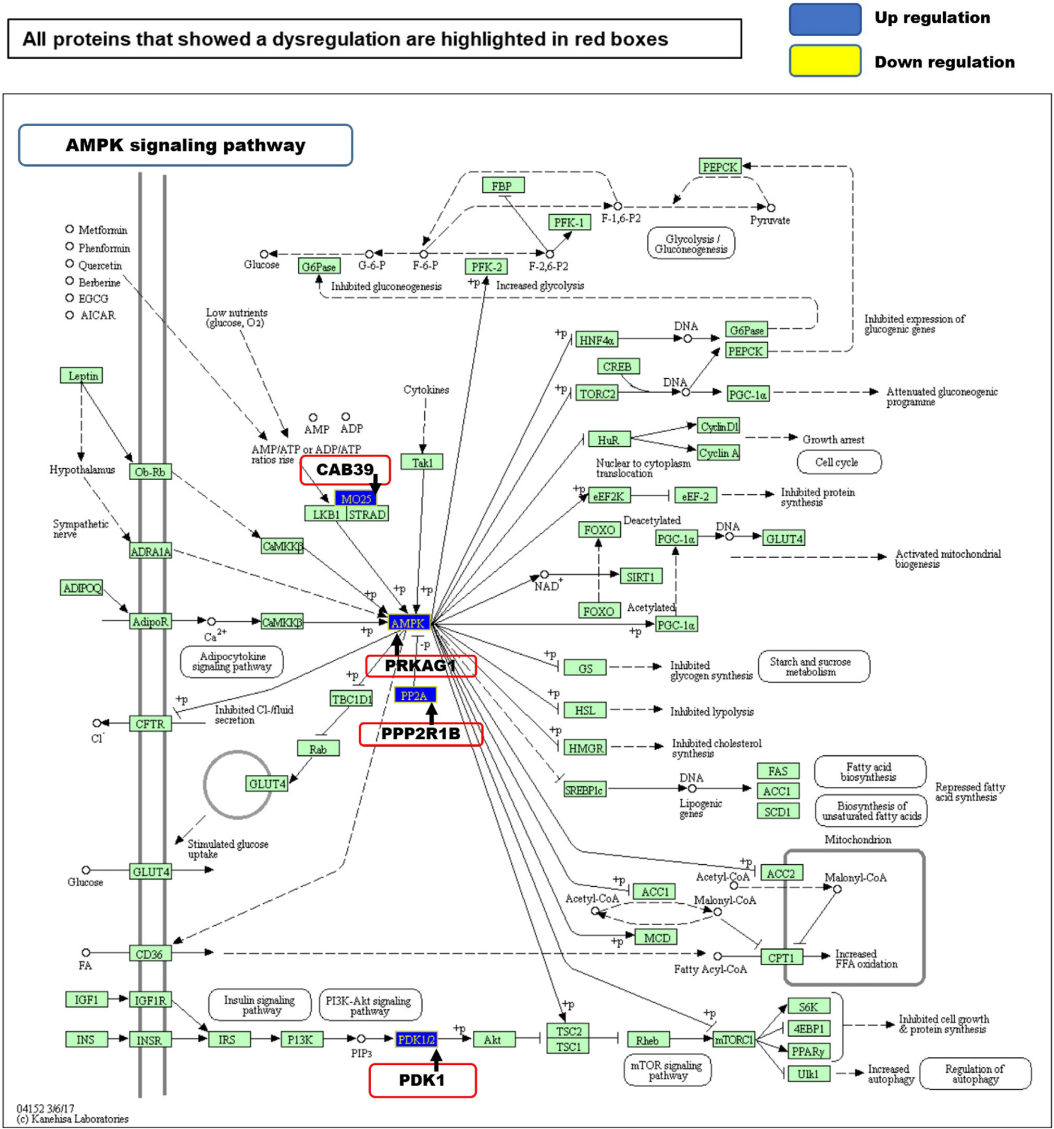
AMPK signaling pathway depicted using KEGG in tissue lysate fraction The levels of upregulation of the proteins observed in AMPK pathway are as follows: CAB39/MO25 log2 0.54-fold, PRKAG1/AMPK log2 0.77-fold, PPP2R1B/PP2A log2 0.77-fold and PDK1 log2 0.56-fold. (Arrow = Protein name, protein complex name or protein function. Red bordered boxes = regulated proteins from the query dataset. +P = phosphorylating events. Blue = up regulation (log2 fold-change) and Yellow = down regulation (log2 fold-change)). Pathway analyzed using KEGG database.

The major pathway regulations identified in this study were identified to be influenced from the AMPK signaling pathway (Figures 6 and S7), selective autophagy (aggrephagy and proteasome pathway) (Figures S8 and S9), NADPH oxidase pathway (Figure S10), OXPHOS (Figure S11), gap junction assembly and degradation, ESCRT, protein processing and folding pathway, vesicle-mediated transport, and lysosome vesicle biogenesis (Figures S9–S13). Most of the proteins and interpreted pathways identified in our study are increasingly being recognised in cardiac pathology,^17^^,^^50^ such as gap junctional remodeling,^51^ mitochondrial-bioenergetics and proteostasis,^17^ NADPH oxidase,^52^ and metabolic derangement caused by oxidative stress.^53^

LC3s are important biomarkers of autophagy. Comparative analysis of LC3-I and II levels found that in AF tissue, levels of LC3-I were almost completely depleted, while no detection of the lipidated autophagic vacuole (AV)-associated LC3-II was observed. This suggests that high levels of autophagic flux may be occurring in the AF tissue, where the efficient clearance of AVs by lysosomes, outpaces the supply of LC3-I. P70 S6 kinase (p70S6K) is activated in a signaling pathway that includes mTOR and dysregulation of the mTOR pathway is implicated in human disease processes.^54^ We further tested by Western blotting the levels of p-p70 (p-P70: Total p70) to provide insights into mTOR activity (Figures S2C–S2E). This was measured using a p- P70 (Thr 389): Total p70 ratio, where Thr389 is an mTOR specific epitope. The p-p70 was unchanged, which suggests the heightened autophagy is not mTOR-dependent (Figures S2C and S2D).

The contemporary transcriptomic and proteome of studied samples may vary substantially in terms of overall correlation between transcript and associated protein. In static cell contexts with minimal temporal dynamics, overall correlation can be quite strong,^55^ but in cells undergoing dynamic response to stimuli or stress, correlation can vary from strongly correlated to almost uncorrelated on a gene by gene basis.^56^ To integrate across proteomic and transcriptomic results, we adopted the method of Cox and Mann^57^ to compare changes in terms of pathway up or down regulation relative to a global median across all pathways with identified members. This approach synthesizes pathway changes across all genes/proteins and therefore allowed for more meaningful comparisons of the implication of changes in the transcriptome versus proteome. The approach revealed that, while there was a general downregulation of pathways related to the EL as scored by mRNA levels, this drop was not consistently reflected by changes in the same pathways as scored by proteins. This is partially reflective of the time lag between response at the mRNA level versus response at the protein level, determined by regulation factors, in particular protein translation and turnover rates that vary from protein to protein.

Bioinformatics analysis of our proteomics results suggests that AMPK upregulation is triggered through an ATP depletion path. V-ATPase proton transporters utilize ATP to conduct protons which acidify the lysosomes or EL (Godbey, 2014), which is pivotal for the lysosomal enzymes to be functionally activated. For example, dysfunctional V-ATPases lead to neurodegenerative disorders^58^ caused by poor substrate digestion in the lysosomes. The increased ATP consumption in tachyarrhythmias,^59^ such as AF, is expected to create an energy demand in the atria.^60^ As the cell’s primary energy source, ATP drives active and coupled membrane ion transport^61^ to maintain cellular ion homeostasis.^62^

### Exploring the functional endolysosomal network proteins in AF

The endolysosomal String network (Figure 3) consists of 134 nodes and 171 edges with an average node degree of 2.55. The functional enrichments in the network were determined using the GO database. 25 proteins from the biological process of intracellular transport and 24 proteins related to peptide transport were identified in the EL network. Six proteins were associated with unfolded protein binding, and 58 proteins were identified for catalytic activity. Furthermore, 48 proteins were components of vesicles, and 11 were components of extracellular vesicles. 118 proteins were part of the anatomical entity of the cell, and 3 proteins were from cellular proteostasis. Notably, 2/8 proteins that regulate the lysosomal storage disorder glycogen storage disease were highlighted in the EL network of AF and 2 more (PYGB and AGL) were significantly upregulated in TL (Figure 3; Table 1).

### Endosomal sorting complex required for transport (ESCRT)

Our analysis suggests lower expression levels of proteins involved in the ESCRT pathway in the AF diseased condition (down-regulation of ESCRT-II complex and its cargo). Proteins such as Ras-related protein Rab-7a (RAB7A), and Vacuolar protein-sorting-associated protein 25 (VPS25), were downregulated by −0.50 and −1.04 log2 fold-change respectively (Table 1). RAB7A, a recruiting protein for the tethering molecules,^63^^,^^64^ plays an essential role in the late endosomes to multivesicular bodies (MVB) transition in the ESCRT pathway. Rab-7 is a common modulator/participant in endocytosis and autophagy.^65^ Moreover, RAB7A participates in the lysosome biogenesis through autophagolysosome formation.^66^ Vacuolar protein-sorting-associated protein 25 (VPS25) is a leading regulatory component of the ESCRT complex-II that sorts the endosomal cargo proteins to the MVB^67^ (Figure 3; Table 1).

Lysosomal alpha-glucosidase (GAA) was log2 0.44-fold upregulated in the EL fraction of AF. GAA functions as an enzyme that degrades glycogen in the lysosome^68^^,^^69^^,^^70^ (Figure 3; Table 1). Studies have indicated that a substantially high recombinant human GAA level is required to minimize the abnormal glycogen storage in the skeletal and cardiac muscles^71^^,^^72^ (Figure 3; Table 1).

1,4-alpha-glucan-branching enzyme (GBE1) is an enzyme that functions as a vital component in glycogen biosynthesis. GBE1 was upregulated by log2 1.67-fold in AF goat model compared to that of sham model (Figure 3; Table 1). To increase the glycogen molecule solubility, GBE1 generates α-1,6-glucosidic branches from α-1,4-linked glucose chains.^73^

### Lysosome motility and motor-protein based transport

Lysosome motility is mediated by motor proteins^74^ such as kinesin and dynein and microtubules.^75^^,^^76^ Multiple biological processes, such as degradation of macro molecules,^77^ cellular homeostasis such as autophagy, signal transduction and metabolic signaling for ATP energy and amino acids, intracellular organelle signaling,^78^^,^^79^^,^^80^ require lysosomes to move and position themselves throughout the cytoplasm.^81^ We identified several motor proteins that are associated with lysosome motility that were upregulated, such as tubulins MACF1 up by log2 1.19-fold, Microtubule-associated protein (MAP4) up by log2 1.05-fold, Tubulin-specific chaperone A (TBCA) up by log2 0.58-fold and we also identified TUBB6 (tubulin beta 6 class V) in EL proteomics fraction and it was found to be downregulated log2 -0.95-fold. Clathrin light chain B (CLTB) was upregulated by log2 1.5-fold in the AF condition. CLTB, along with Vesicle-associated membrane proteins (VAMPs) and adaptor protein complex 1 (AP1) play an essential role in lysosome membrane biogenesis^82^ (Figure 3; Table 1). Dynein light chain roadblock-type 1 (DYNLRB1) was upregulated by log2 0.98-fold. DYNLRB1 is an essential protein for general dynein-mediated transport and has been shown to be vital for sensory neuron survival.^83^ Furthermore, DYNLRB1 links dynein with adaptor proteins to regulate dynein and cargo for ideal cellular vesicle transport.^84^ Simultaneously, Cytoplasmic Dynein 1 acts as a motor protein for the intracellular motility of organelles and retrograde motility of vesicles along the microtubules^85^ (Figure 3; Table 1). WDR78, a Dynein-f associated motor protein required for the axonemal localization^86^ was upregulated by log2 1.8-fold. Nestin (NES, upregulated by log2 0.96-fold in AF goat) is an intermediate filament (IFs) involved in vesicle-based communication, vesicle interaction and trafficking.^87^ Similarly, vesicle-associated membrane protein-associated protein A (VAPA), a protein involved in vesicle trafficking, was upregulated by log2 0.93-fold in the AF goat model^88^ (Figure 3; Table 1) and is a major ER-lysosome anchor protein.^89^ Meng Lu et al.^90^ showed that abolished VAPA-mediated anchoring compromises ER remodeling and significantly increases the speed of lysosome motility. Eps15 homology (EH) domain-containing protein 1 (EHD1) was upregulated by log2 0.6-fold in the EL fraction. EHDs play critical roles in endosome-based membrane protein targeting.^91^ EHD1 is a retrograde trafficking mediator and a regulator of the cluster of differentiation 44 protein (CD44) that participates in endocytic recycling and lysosomal degradation.^92^^,^^93^ Gudmundsson et al.^91^ found modulation of EHD expression during myocardial infarction, which suggests that these proteins may play important roles in regulating membrane excitability^91^ (Figure 3; Table 1).

### Intermediate protein networks between the whole cell and endolysosomes - Proteasome and aggresome pathway

The proteasome pathway is part of the selective autophagy process^94^ and several dysregulated proteins in our EL disease fraction were from this pathway. Protein degradation through ubiquitination is termed as proteasome or aggresome pathway. Aggregated and misfolded proteins are identified by chaperone proteins and tagged by ubiquitination before protein degradation. Proteins related to both aggresome and proteasome formation were found to be up- or downregulated in our AF samples, including; E3 ubiquitin-protein ligase PPP1R11 (PPP1R11) up by log2 1.7, tubulin α chain (TUBA4A) up by log2 1.09, ubiquitin carboxyl-terminal hydrolase 5 (USP5) up by log2 0.39, probable ubiquitin carboxyl-terminal hydrolase FAF-X (USP9X) up by log2 1.00-fold, tubulin β chain (TUBB2A) down by log2 -1.07-fold (Table 1 and Table S1.2). PPP1R11 and USP5 are ubiquitination triggering proteins, and USP9X is both a deubiquitinase, that prevents a protein from the removal of conjugated ubiquitin and a ubiquitin precursor processor^95^^,^^96^(Table 1). The upregulated aggresome formation process that we observed in AF goat model is a well-known factor for the changes in lysosome distribution and its motility.^97^ Furthermore, Chaperonin containing TPC1 subunit 2 (CCT2) functions independently of ubiquitin and the TRiC complex to facilitate autophagic clearance of solid protein aggregates.^98^ We detect modestly upregulated levels log2 (0.55) of CCT2 in the EL fraction. In addition to CCT2, we also observe upregulated levels log2 (1.00) of CDC37 and log2 (0.48) COPS7A in the EL fraction. Hsp90-CDC37 complex appear to participate in upstream of autophagy activation for the control of protein quality,^99^ and COPS7A is a protein from the COP9 signalosome complex (CSN) that mediates de-neddylation to regulate the ubiquitin conjugation pathway and is presumed to participate in structural remodeling that plays a crucial part in the developing stages of AF.^44^

### AMPK upregulation

AMPK pathway is a central regulator of cellular metabolism that is activated mainly by reduced adenosine triphosphate (ATP) levels in the cell (Figures 6 and S7) and cascades a series of downstream chemical reactions in the cell that reprogram metabolism, autophagy,^100^ cell polarity and growth^101^ As a result, catabolic pathways are upregulated while inhibiting the anabolic pathways to reduce the cellular ATP consumption level.^102^

Our Integrated proteomic and transcriptomic data showed a significant upregulation in AMP-activated protein kinase (AMPK) pathway related proteins in AF samples. 5′- AMP-activated protein kinase subunit gamma-1 (PRKAG1/AMPK) log2 0.8--fold, calcium-binding protein 39 (CAB39/MO25) log2 0.54 -fold, Serine/threonine-protein phosphatase 2A regulatory subunit A beta isoform (PPP2R1B/PP2A) log2 0.77-fold and Protein-serine/threonine kinase (PDK1) log2 0.56-fold from AMPK pathway were observed to be upregulated in AF samples (Figures 1B, 6, S7, and Table S1.2). These observed changes suggest a link between AMPK regulatory pathway protein upregulation and AF.^19^^,^^103^^,^^104^

The identified AMPK pathway proteins are from AMPK activation through the ATP pathway. CAB39/MO25 is part of the liver kinase B1 (LKB1) and STE-related adaptor protein STRAD protein complex,^105^ which binds and activates STK11/LKB1. AMPK protein activity is controlled by LKB1, acting as a key upstream regulator for AMPK phosphorylation. PPP2R1B assembles the catalytic subunits of AMPK, and signals from insulin to PKB/AKT1 are transduced by PDK1 through activated phosphorylation. This downstream signaling cascade targets cell survival, glucose and amino acid uptake and glucose storage.^106^ Therefore, processes such as upregulation of ATP production, activation of glucose intake, inhibition of the cell proliferation and growth, autophagy activation, cytoskeletal remodeling, DNA damage response, and apoptosis^102^ are regulated by AMPK. The increased expression of the CAB39, AMPK, PP2A and PDK1 proteins provides further support for the upregulation of the AMPK signaling pathway in AF. Of interest is the finding by,^107^ related to molecular mechanism of electrical modeling, where they showed an increased activity of PP2A (protein phosphatase 2A) results in hypophosphorylation of the calcium channel *I*_*CaL*_ (see also Review by.^108^

### Ras-homologous guanosine triphosphatases (Rho GTPases) activation of Nicotinamide adenine dinucleotide phosphate oxidase (NADPH oxidase)

We observed increased levels of proteins from the MAP Kinase signaling network, which represent the RHO-GTPase activation of NADPH Oxidase. These regulated proteins are: Mitogen-activated protein kinase 3 (MAPK3) up by log2 1.37-fold; Mitogen-activated protein kinase 12 (MAPK12) up by log2 0.51-fold; Mitogen-activated protein kinase 14 (MAPK14) up by log2 0.42-fold; and Protein S100-A9 (S100A9) up by log2 1.17-fold (a complete list of the most significantly regulated TL proteins identified in the AF goat model are provided in Table S1.2). MAPK proteins are reported to conduct signaling in mitochondria, Golgi, endoplasmic reticulum (ER) and endosomes.^109^ Rho family small GTPase proteins activate NADPH Oxidase (NOX), which activates the leading cell stress-response signaling network MAPK (see integrated omics Figures S4B, S5B, S7, and S10). NOX signaling is a cellular stress-responsive mechanism in the cardiovascular system,^110^^,^^111^ causing the production of superoxide, a reactive oxygen species, during cellular stresses initiated by biological, physical or chemical triggers. Cytochrome *b*-245 heavy chain (CYBB) or NADPH Oxidase 2 is a membrane-bound enzyme that generates superoxide (upregulated by log2 2.06-fold change in our transcriptomics data, a complete list of the most significantly regulated genes identified in the AF goat model mRNA analysis are provided in Table S1.1). The Yoo S et al. 2020 study showed that the oxidative injury by the CYBB/NOX2 caused an electrical remodeling by upregulating the constitutively active acetylcholine dependent potassium current (IKACh) in the canine model.^112^ Significantly higher NOX-induced superoxide levels have been observed in AF patients, and unlike NOX2, the superoxides produced by dysfunctional NOX had a lesser contribution to electrophysiological remodeling and oxidative injury in the atria of AF patients,^52^ and these superoxides further worsen the disease condition.^113^^,^^114^^,^^115^^,^^116^

MAPK3 is a signal transducing protein that regulates transcription, translation,^117^ and cell cycle-related functions such as the arrangement of the cytoskeleton and cell-cell adhesion during the cell survival state.^118^^,^^119^ Furthermore, MAPK/ERK participate in lysosomal dynamics^120^ and endosomal recycling.^121^ MAPK12 is another signal transducing protein that, is triggered by extracellular stress stimuli, such as pro cytokines^122^ (Figure S10).

MAPK14 is also stimulated by inflammatory triggers^123^ that conduct the cellular protein turnover for degradation through proteasomes,^124^ and S100A9 is a calcium- and zinc-binding protein that plays a prominent role in the inflammatory and immune response.^125^ The observed protein changes in this pathway, indicate increased cellular stress status in our AF goat model (Figure S10) highlighting the relevance of these findings relating to the emerging role of NOX pathways in AF.^113^

### Identifying differential mRNA expression by quantitative transcriptomic analysis

We performed transcriptomics as a confirmatory and supplementary method to our proteomics screen in our goat AF model (Figure S4A). The transcriptome approach has allowed for the detection of unbiased molecular changes in AF.^126^ There is evidence for inter-related pathways such as oxidative stress, inflammation, thrombogenesis and fibrosis^126^ and more recently autophagy.^127^ Our transcriptomic analysis identified upregulation of major ion channel Potassium/sodium hyperpolarization-activated cyclic nucleotide-gated channel 1 (HCN1) by log2 2.80-fold (Table S1.1).^128^^,^^129^ HCN1 gain of function promotes AF. Furthermore, Glycogen debranching enzyme (AGL) was upregulated by log2 2.85-fold change (Table S1.1).

Reactome analysis^130^ was performed on the differentially expressed genes. The most significantly regulated proteins and genes are presented in a hierarchical visualization of pathways using space filling graphs in Figures S16–S18. KEGG pathways of proteomics as well as transcriptomics and integrated Reactome pathway analysis highlighted AMPK signaling pathways as significantly upregulated (Figure 6, Table S1.1 and a complete list of pathways from the integrated EL, TL proteomics and transcriptomics analysis is in, Table S2.1, and the most significantly highlighted pathways of EL fraction and TL from Reactome analysis are shown in Tables S2.2 and S2.3), and Ribosome biogenesis as significantly down regulated (Figure S14). AMPK (upregulated): These include the following 8 genes SLC2A4, PFKFB2, PRKAA2, STRADB, CAB39L, PRKAG3, PPP2R3A and CCND1. Ribosome biogenesis (downregulated): These include the following 8 genes TRMT112, TBL3, GNL3, GAR1, FBL, NOP56, POP1 and NOB1 (Table S1.1).

Reactome allows us to overlay our quantitative expression data to visualize the extent of change and progression in affected pathways. In the mRNA analysis we found 231 out of 380 identifiers and 885 pathways were identified by at least one identifier (Figure S18). Table S2.4 shows the 25 most relevant pathways sorted according to the *p*-value.

In summary, the analysis highlights rRNA processing (down regulations), major pathway of rRNA processing in the nucleolus and cytosol (down regulations), unfolded protein response (UPR, down regulations), metabolism of RNA (down regulations), and interferon signaling (up regulations). AMPK, as well as being the master regulator of energy homeostasis, is also a physiological suppressor of UPR.^131^

There is growing evidence linking AF to metabolic stress and inflammation.^132^^,^^133^ In the cell, ribosomes control translation of proteins and their activity accounts for most of the cells energy consumption.^134^ Ribosome biogenesis depends on the nutritional and energy status of the cells and is vulnerable to internal and external stress stimuli. Impaired ribosome biogenesis (e.g., in aged tissue) may be protective or a compensatory mechanism.^135^ AMPK is a central regulator of energy homeostasis^136^ playing a key role in monitoring cellular energy metabolism and there is growing evidence around the importance of AMPK in the heart.^132^^,^^137^^,^^138^ Recently Cao et al.^139^ showed that γ2-AMPK translocate into the nucleus to suppress pre-rRNA transcription and ribosome biosynthesis during stress, this reduces ER stress and cell death. We identified PRKAG3 (5′-AMP-activated protein kinase subunit gamma-3) among our upregulated genes (upregulated by log2 2.31-fold, Table S1.1). This seems particularly interesting in light of the findings by Cao et al.^139^ where activation of γ2-AMPK suppresses ribosome biogenesis and protects against myocardial ischemia/reperfusion injury. Our transcriptomics data suggests an adaptation process to the stress conditions created during AF. AMPK signaling is increased, and this consequently would be expected to affect ribosomal RNA transcription, as reflected in the genes that are upregulated (Table S1.1) Su et al.^132^ highlight a critical role played by AMPK signaling and the resultant alterations in electrophysiological function and structural remodeling in the atria. Additionally, Su et al.^132^ highlight loss of AMPK affecting the expression of mRNA transcripts encoding gap junction proteins and ion channels in the atrium. Here, our goat model aligns with the notion of an upregulation of AMPK to downregulate energy-consuming processes like ribosome biogenesis.

The datasets obtained, and shared in this study offer a much wider scope for molecular signaling analysis in the goat AF model. Other aspects of AF and aging could be explored in our datasets. As recently was shown in^140^ that reducing the speed of RNA polymerase II by overexpressing histone components (which counter age-associated changes in nucleosome positioning), extended lifespan in flies and the division potential of human cells. We note in our study POLR2E (major component of Polymerase II complex) downregulation in transcriptomics and histone H3 upregulation was found to be upregulated in proteomics (Tables S1.1 and S1.2). This observation lends weight to our hypothesis that at this six month stage of AF in the goat model, organelle function seems to push in the direction toward compensation mechanisms to favor pro-survival modes.

### Glycogen accumulation and lysosomal GAA upregulation

Histological studies in healthy/normal goat by Embi et al. 2014 showed left atrial appendage glycogen levels always exceeded right atrial appendage levels.^141^ The density and location of glycogen was also distinct and suggested these differences in glycogen were a potential contributory mechanism for the initiation and maintenance of AF, particularly the greater propensity for developing an AF substrate in the left versus the right atrium.^141^ Studies by Zhang et al. 2015 in pacing-induced AF (dog model), showed AF promoted glycogen deposition.^142^ In our proteomic data, significantly regulated proteins in the LA of AF goat included GLUT4/SLC2A4, a protein that transports glucose inside the cell (upregulated by log2 1.55) (Tables S1.2 and S2.4). The GLUT4 vesicle translocation to the plasma membrane (Figures S7 and S12) is conducted by the tethering and docking proteins Caveolin (CAV1) and Vesicle-fusing ATPase N-ethylmaleimide-sensitive fusion protein (NSF).^143^ We observe a significant upregulation of NSF by log2 1.55 (Figure 1B and Table S1.2) and CAV1 by a fold-change of log2 0.57 (Figure S12 and Table S1.2). Moreover, lysosomal-α-glucosidase (GAA), the glycogen degrading enzyme in the lysosomes,^68^^,^^69^ was upregulated by a log2 fold-change of 0.44 in the EL fraction of the AF goat model (Figure 3; Table 1). We chose to quantitatively assess the levels of Glycogenin 1 (GYG1) because in eukaryotes, Glycogenin 1 enzyme initiates glycogen biogenesis by producing an oligosaccharide primer that functions as a substrate for glycogen synthesis in bulk.^144^ Western blotting was performed on the enzyme GYG1 and a trend towards (*p* = 0.007) upregulation of GYG1 was observed in the AF samples.

Figures S2F and S2G, provide evidence for increased glycogen synthesis in the AF goat model.^141^ Here we present specific protein changes that impact on glycogen levels in the cells (Figures S2F and S2G). Reassuringly, the evidence presented from our whole tissue omics analysis (Figures S4B, S5B, and S7), is in keeping with existing structural data (canine^142^ and goat AF model^22^) linking glycogen accumulation and fibrosis as factors in the persistent forms of AF.^22^

### Increased autophagic flux in AF goat model

Western blotting was performed on sham and AF groups to identify effects of AMPK upregulation in autophagy (Figures S2A and S2B). For the upregulation of autophagy flux LC3I, and for impaired autophagy flux, LC3II protein markers were blotted. A depletion of LC3I was detected by the absence of LC3I protein in AF compared to the sham goat model, suggesting the upregulation of autophagy flux. Moreover, the LC3II protein was absent in both sham and AF goat models suggesting the absence of impaired autophagic flux instead pointing toward the presence of an overactive flux. This result aligns with a recent study by.^18^ Network analysis shown in Figure S7, which presents a close network interaction of AMPK with lysosomal and vesicle localized proteins Ras-related GTP-binding protein A (RRAGA), 1,4-alpha-glucan-branching enzyme (GBE1), Glycogen debranching enzyme (AGL), Phosphoglucomutase-1 (PGM1), and Glycogen phosphorylase, brain form (PYGB).

Our integrated omics analysis comparing EL proteomics and transcriptomics highlights important changes occurring in EL proteins involved in this chronic goat AF model. We see a downregulation in suppression of autophagy, MAPK1 activation, inhibition of nitric oxide (NO) production, mitochondrial electron transport chain deregulation, changes in inflammation status (Interleukins), glycogen disease-like pathologies, changes in gap junction activity, stimulation of cell death response by PAK-2p34, upregulation of RhoBTB proteins that are involved in vesicle trafficking processes and retrograde transport from endosomes to the Golgi apparatus (Figure S4B integrated omics). Our observations are relevant and fit with many observations published over the years including the findings that human atrial samples from patients with AF have increased immune cell infiltration compared to those from patients without AF.^145^ Additionally, NO produced by endothelial NO synthase (eNOS) plays a role in the regulation of cell growth, apoptosis, and tissue perfusion^146^ and our findings of apoptosis and downregulation of NOS corroborates early findings.^147^ ER stress and oxidative stress have been highly implicated in many cardiac pathologies^148^^,^^149^ including the pathogenesis of AF.^150^ Recent studies highlight the importance of PAK2. In Pak2 cardiac deleted mice under stress or overload, there is a defective ER response, cardiac dysfunction, and profound cell death.^151^

In recent years, a complex relationship between AF, systemic inflammation and oxidative stress has come to light.^152^ Some evidence suggests that the underlying atrial changes that lead to the development and progression of AF may be inflammatory in nature.^153^ Histological examination of atrial tissue taken from patients with so-called 'lone' AF refractory to conventional antiarrhythmic treatment showed that two-thirds (66%) had inflammatory changes significant enough to be classified as myocarditis when compared with control samples from patients with Wolff-Parkinson-White syndrome.^7^ Patients with some systemic autoimmune disorders such as rheumatoid arthritis are at increased risk of developing AF^154^ and the severity of inflammation as measured by levels of C-reactive protein (CRP) correlates with the incidence of AF in conditions characterized by chronic inflammatory changes.^155^ It has also been observed that the apogee of the surgical stress response usually occurs at approximately 72 h post-surgery which coincides with the peak post-operative incidence of new-onset AF.^156^ Moreover, recent work using data from the UK Biobank has a shown strong association between AF and inflammatory indicators in nearly half a million patients.^157^

Some anti-inflammatory agents have been associated with a reduction in AF burden. One meta-analysis demonstrated a reduction in post-operative AF in patients following cardiac surgery when glucocorticoids were given,^158^ and in a small trial with patients who had AF catheter ablations, a short course of steroids given after the procedure led to a reduction in early (although not late) recurrence of AF.^159^ Other agents with anti-inflammatory properties, such as statins and colchicine have also been associated with a protective effect against AF.^160^^,^^161^^,^^162^

Our findings lend weight to these conclusions and provide further evidence for the molecular changes in metabolism that may underpin the development of AF. Out of the 2104 proteins, 340 proteins in TL and 148 in EL were significantly changed in AF. We validated Rab11 (Ras-related protein 11) using Western blotting. The LC3I protein was absent in AF, suggesting an increased autophagic flux. The TL fraction, highlighted mitochondrial oxidative-phosphorylation (OXPHOS) and AMPK pathway protein upregulation, indicating a potential increased ATP energy demand in AF. The EL proteins GAA, Rab7a, CLTB, VPS25 and CCT2 were significantly regulated. The upregulation of protein processing suggests increased vesicular trafficking, potentially related to increased metabolic energy demands. Here we use an endolysosomal purification protocol to study atrial specific protein changes in AF and noted changes in EL proteins. We believe this approach allows us to study differences in protein expression more specifically related to EL and globally in health and disease. In order to provide further direct experimental confirmation, our data is expected to provide rationale for future experiments that study links between the EL and AF. These may include viral vector-RNA overexpression in rodent or human induced pluripotent stem cell (iPSC) derived cardiomyocytes targeting the regulated genes and proteins identified in our goat AF omics analysis. As an example, oxidative stress and the AMPK pathway can be induced by hydrogen peroxide and lowering ATP via reduced glucose supplementation to upregulate the AMPK pathway, targeting downstream autophagic pathways and increasing vesicle trafficking of endolysosomal proteins such as GAA, Rab7a, CLTB, VPS25, CCT2 and related proteins. With the availability of AF patient derived cardiomyocytes differentiated from iPSCs,^163^ electrophysiological and pharmacological approaches might be useful to test any direct or indirect link between AF and EL. Measuring cathepsin activity as a readout of endolysosomal signaling in AF is another experimental option in the future. Other experimental techniques to explore the involvement of lysosomes in AF include live imaging of isolated human atrial cells to study the pH of whole cell or EL in AF cells vs. sinus rhythm controls with ratiometric dyes such as OG488. Endolysosomal related gene knock-in or knock-down (using endolysosomal hits identified in this study) approaches in iPSC cardiac models can also be used to demonstrate whether they can induce functional alterations leading to a cardiac substrate more prone to develop arrhythmias under demanding conditions (e.g., adrenergic stimulation), helping to dissect cellular mechanisms underlying AF.

### Conclusion

Studies have shown that prominent differences between paroxysmal AF and sinus rhythm patients relate to changes in expression of proteins involved in metabolic processes.^164^^,^^165^ Ozcan et al. 2015 provided evidence into the role of atrial metabolism for AF substrate evolution, findings that are relevant when considering alternative therapeutic approaches to prevent AF progression.^166^ Our EL organelle omics approach helps us describe a disease setting where there is increased cellular stress, increased vesicle trafficking, changes in ATP demands, accumulation of glycogen, inflammation, and stimulation of cell death. Our findings uncover new insights linking endolysosomal proteins, ER stress response, RNA biogenesis, and cell apoptosis pathways which may be triggered by failure of protective ER stress response. The current pharmacological therapies for AF are not sufficiently effective to control disease progression and new molecular insights can fuel the development of novel therapeutic strategies. EL in the heart provide a significant contribution to basal calcium transient amplitude and beta-adrenergic responses in both atrial^167^ and ventricular^79^ myocytes. Clearly, the multi-functional role of the EL appears to be at play in this disease setting and these results highlight the need for further investigation into the role of endolysosomal pathways in cellular dysfunction and apoptosis in AF. In summary, our endolysosomal proteomics and integrated omics analysis pave the way for future studies focused on identifying suitable EL targets, drug discoveries and biomarker identification. The information provided present a promising option for exploring new pathways for the treatment of AF.

### Limitations of the study

The molecular pathways applied on the protein regulations found in this project are interpretations of published literature and databases and provide a fundamental understanding of the disease pathways. Therefore, an establishment of the protein regulation as a complete pathway alteration needs further and extensive investigation. We have used a large animal goat AF model in which chronic AF was maintained by pacing for six months. The data we present is a snapshot of genes and proteins relevant at that point in time of the disease. The multifactorial and heterogeneous nature of AF as a disease^25^^,^^168^^,^^169^ pose limitations in the assessment of the occurrence and changes in gene and protein expression especially during the process of disease progression from paroxysmal to chronic AF.

## STAR★Methods

### Key resources table

**Table:** 

REAGENT or RESOURCE	SOURCE	IDENTIFIER
**Antibodies**		

Anti-GYG1	ThermoFisher Scientific	Cat# PA5-116508; RRID: AB_2901139
Anti- Lamp2	ThermoFisher Scientific	Cat# PA1-655; RRID: AB_2134625
Anti-COX IV	Abcam	Cat# ab16056; RRID: AB_443304
Anti-Phospholamban	Abcam	Cat# ab85146; RRID: AB_10974942
Polyclonal Goat Anti-Rabbit Immunoglobulins/HRP	Agilent Dako	Cat# P044801-2; RRID: AB_2617138

**Biological samples**		

Left Atrial tissue from *C. hircus* Atrial fibrillation models	Maastricht University, Netherlands	

**Chemicals, peptides, and recombinant proteins**		

Lysosome Isolation Buffer	BioVision	K235-50-1
Lysosome Enrichment Buffer	BioVision	K235-50-2
Protease Inhibitor Cocktail	BioVision	K235-50-4
Percoll	Santa Cruz Biotechnology	sc-500790
Sucrose	Fisher Scientific	15503022
4-Methylumbelliferyl N-acetyl-b-D-glucosaminide	Merck (Sigma-Aldrich)	CAS 37067-30-4
Na_2_CO_3_	Merck (Sigma-Aldrich)	CAS 497-19-8
Bolt™ 4 to 12%, Bis-Tris, 1.0 mm, Mini Protein Gel, 10-well	ThermoFisher Scientific	NW04120BOX
MES SDS Running Buffer	Life Technologies	NP0002
Transfer Buffer	ThermoFisher Scientific	NP0006
Westar Supernova detection substrate	Cyanogen	XLS3,0020
Triton X-100 solution	Merck (Sigma-Aldrich)	CAS 9002-93-1
Sodium Acetate Buffer	Merck (Sigma-Aldrich)	CAS 126-96-5
Tris HCL	Merck (Sigma-Aldrich)	CAS 1185-53-1
Iodoacetamide	Merck (Sigma-Aldrich)	CAS 144-48-9
Dithiothreitol	Merck (Sigma-Aldrich)	CAS 3483-12-3
Urea	Merck (Sigma-Aldrich)	CAS 57-13-6

**Deposited data**		

Mass spectrometry proteomics data, Proteome Xchange via PRIDE partner repository, dataset identifier PRIDE: PXD041056	This paper	http://proteomecentral.proteomexchange.org/cgi/GetDataset?ID=PXD041056
mRNA sequencing Transcriptomics data, GEO Accession code: GSE228289	This paper	
A modified density gradient proteomic-based method to analyze endolysosomal proteins in cardiac tissue	Ayagama et al., 2021^21^	https://www.cell.com/iscience/fulltext/S2589-0042(21)00917-2

**Experimental models: Organisms/strains**		

*C.hircus*	Maastricht University, Netherlands	N/A

**Software and algorithms**		

Progenesis QI software platform (version 4.2)	Waters™ Cooperation	www.nonlinear.com
Perseus software platform (version 1.6.15.0)	Tyanova et al., 2016^170^	http://coxdocs.org/doku.php?id=perseus:start

### Resource availability

#### Lead contact

Rebecca-Ann Burton. r.a.b.burton@liverpool.ac.uk.

#### Materials availability

This study did not generate new unique reagents or codes.

#### Data and code availability

Mass Spectrometry data has been deposited in PRIDE and genomics data in GEO. See resource availability for accession numbers. Data can be requested by contacting the lead contact. This study did not generate new unique codes.

### Experimental model and study participant details

#### Animals

AF was induced and maintained in farm reared female goats (C. hircus) aged between 24 and 34 months (weight 72 ±8 kg) for 6 months (AF goat model was created as conducted in,^42^ followed by an open chest sacrifice experiment (*N* = 4 AF and *N* = 4 sham controls, N = each animal, n = the number of replicates from each condition). (The goat study was carried out in accordance with the principles of the Basel declaration and regulations of European directive 2010/63/EU, and the local ethical board for animal experimentation of the Maastricht University approved the protocol).

### Method details

#### Tissue homogenization

Frozen left atrial tissue biopsies of AF and sham goat were thoroughly cleaned using phosphate buffered solution (PBS) and weighed. A minimum of 100 mg tissue is weighted in order to perform proteomics. Each atrium biopsy sample was cut using sterile scalpels and gently homogenized using a 7 mL Dounce homogenizer in Lysosome isolation buffer (LIB) [Containing 1:500 protease inhibitor cocktail (PIC) and phosphatase inhibitor (PHI) (Bio vision), (PhosSTOP Roche)]. Preparations were further homogenized in a 1 mL Dounce homogeniser and transferred to chilled 1.5 mL ultracentrifugation tubes (Beckmann coulter). Sample preparations were mixed at a ratio of 1:1.5 Lysosome enrichment buffer [(LEB) (Biovision, containing 1:500 PIC)] to homogenate by inverting tubes, and were stored on ice for 5 min until the centrifugation.^21^

##### Tissue lysate (TL)

Samples were centrifuged at 13,000 g × 2 min at 4°C (TLX Beckmann Coulter Ultra Centrifuge) and the supernatant or the TL, was collected.^21^

##### Endo-lysosome fraction (EL)

The collected supernatant was retained and repeated for a further centrifugation step at 29,000 g × 30 min at 15°C (500 μL of under-laid 2.5 M sucrose with over-laid 500 μL Percoll). The supernatant above the sucrose and Percoll intermediate was collected for further fractionation. Firstly, ultracentrifuge tubes were underlaid with 2.5 M sucrose and overlaid with a series of Percoll dilutions (1.11 g/mL – 1.04 g/mL in ddH2O). The ultracentrifuge tubes were centrifuged at 67,000 g × 30 min at 4°C. The fraction at 1.04 g/mL was collected and labeled as the endolysosomal fraction (EL).^21^ *N* = 3 biological replicates for each AF and sham conditions were used in proteomic analysis.

#### Liquid chromatography-tandem mass spectrometry analysis

5 μL of 200 mM dithiothreitol (30 min at room temperature) was added to each sample to reduce and proceeded with alkylation using 20 μL of 200 mM iodoacetamide (30 min at room temperature), followed by methanol-chloroform precipitation. The sample proteins were pelleted at this stage and was re-suspended in 6 M urea in 400 mM Tris-HCl, pH 7.8. The 6M urea was then diluted to 1 M with 400 mM Tris-HCl at pH 7.8, and the proteins were digested in trypsin at a ratio of 1:50 (overnight at 37C°). The trypsin digested samples were then processed at the Target Discovery Institute, Oxford.

Samples were then acidified to a final concentration of 1% formic acid, and the samples were desalted on Sola HRP SPE cartridges (Thermo Fisher Scientific) and dried down using a SpeedVaccum centrifuge. Dried down protein Samples were further desalted online (PepMAP C18, 300 μm × 5 mm, 5 μm particle, Thermo Fisher Scientific) for 1 min (flow rate of 20 μL/min and separated on an EASY-Spray column) (PepMAP C18, 75 μm × 500 mm, 2 μm particle, ES803, Thermo Fisher Scientific) over 60 min using a gradient of 2–35% acetonitrile in 5% DMSO/0.1% formic acid at 250 nL/min. Separation and analysis were performed on a Dionex Ultimate 3000 RSLC system coupled to an Orbitrap Fusion Lumos platform (both Thermo Fisher Scientific) using standard parameters (Universal Method).^171^

MS scans were acquired at a resolution of 120,000 between 400 and 1,500 m/z. An AGC target of 4.0E5 and MS/MS spectra detection was carried out using rapid scan mode in the linear ion trap at a 35% collision energy after collision-induced dissociation fragmentation (CIDF). An AGC target of 4.0E3 for up to 250 ms, employing a maximal duty cycle of 3 s, prioritising the most intense ions and injecting ions for all available parallelisable time. Selected precursor masses were excluded for 30 s.^171^

Mass spectrometry data were analyzed quantitatively with the Progenesis QI software platform (WatersTM Cooperation, www.nonlinear.com) (version 4.2), and database searches were carried out against the UniProt C. hircus database (UP000291000). Automatic processing was selected. All runs in the experiment were adjusted to the function suitability, and runs were aligned automatically. The peak picking was selected between 10 and 75 min. The group runs option was set to conditions, and relative quantitation using Hi-N was selected. Finally, proteins were grouped.

### Quantification and statistical analysis

#### Quantification and statistical analysis of mass spectrometry data

Quantitative analysis for significant differences of protein regulation between the AF and sham conditions of TL and EL samples and data visualization were performed using the Perseus software platform^33^ (version 1.6.15.0). Using protein intensity values of biological replicates, the protein groups were created and uploaded as a data matrix in Perseus with the respective protein abundances as main columns. The data matrix was reduced by filtering based on categorical columns to remove proteins where more than two intensity values were absent from six biological replicates, and remaining data with no more than 2 missing values were quantitatively analyzed. A total of 2,104 proteins in TL and EL remained after filtering. Groups of biological replicates for TL and EL fractions were defined in categorical annotation rows. Data were log transformed (log2) and normalised via *Z* score.

Data imputation was not required due to Progenesis reporting signal noise in absence of peptide precursors (Tables S4 and S5). Therefore, principal component analysis (PCA) was performed on 100% valid values. A volcano plot was generated based on normalised intensities applying two-way Student’s t test to probe for significant difference of protein regulation between AF and sham conditions of each TL and EL samples. A permutation-based false-discovery rate (FDR) was determined with 250 randomizations and S0 = 0.1 (default). The quantified proteins were accounted with 99% confidence level at 5% FDR.

##### Principal component analysis (PCA)

Principal component analysis is a reduced data dimension-interpretation of the protein groups’ distribution between the sample groups and it is one of the most popular multivariate statistical techniques. A PCA plot can provide a window to a large dataset by identifying the common vectors, therefore summarizing the variation.^172^ As indicated in Figure 2E the respective component 1 and component 2 vector deviations were observed between AF (purple symbols) and sham (green symbols) EL groups demonstrating that the variability is driven by the differential experiment groups rather than the variability within the sample group.

##### Violin plot analysis

The violin plots were produced using the InstantClue omics tool.^173^ The protein intensities used were transformed to log2, normalised by z scoring, and AF vs. sham groups were colour-coded; a gradient of purple for the distribution of protein intensities (Figure 2A). This Violin plot represents a kernel density estimation of the underlying protein intensity distribution (The kernel estimated density distribution is the nonparametric representation of the probability density function of a random variable (www.mathworks.com). The quantified protein intensity matrix used for the violin plot is created in Perseus 1.6.15.0 ^33^.

#### Heatmap analysis

The heat or clustering maps were produced in InstantClue omics tool using Euclidian distance and K-mean clustering of the normalised protein intensities. The intensity values are obtained from the quantified matrix created in Perseus 1.6.15.0. A total of 148 proteins in EL were categorized into protein clusters according to the protein intensities (EL = 3 clusters). The regulation level variations are displayed using color codes (red and blue), where red represents the up regulation of the protein clusters and blue represents the down regulation of these clusters (Figure 2C).

#### Statistics for network analysis

The string network edges of EL and TL were created based on the interaction evidence, which are experiments, gene fusions, databases, co-occurrences and co-expressions. The minimum required interaction score was set to default or medium confidence (0.4), and only the query proteins were used and external interactors were excluded.

#### Sample preparation for transcriptomics

Frozen AF and sham LA goat tissue were collected without the RNase contamination, and samples were thawed using an RNAlater stabilisation solution (AM7020, Invitrogen). Samples were then homogenised using bead disruption, and in a 1 mL of Trizol (15596026, Thermo Fischer), approximately 50–100 mg of tissue were solubilised. The supernatant was collected from lysates after incubating them in RT for 5 min and centrifuging at 12,000 × g for 5 min at 4°C. Furthermore, it was followed with a re-centrifugation at 12,000 g at 4°C for 15 min, after vigorously shaking with 200 μL chloroform. The upper aqueous phase was separated, 1:1 volume ice-cold isopropanol was added, and gently mixed. This procedure was followed by centrifugation at 12,000 g for 30 min at 4°C, and the collected RNA pellet was washed with 75% ethyl alcohol (10048291, Thermo Fisher) and dried.

#### Qualification and quantification of mRNA

The isolated RNA was screened for contaminants and degradation levels using 1% agarose, and a NanoPhotometer spectrophotometer (IMPLEN, CA, USA) was used to check the purity (RNA Nano 6000 Assay Kit of the Bio-analyser 2100 system (Agilent Technologies, CA, USA) was used to detect the RNA integrity and assess quantitation.

Sequence libraries were prepared using the NEBNext Ultra TM RNA Illumina (NEB, USA) Library Prep Kit. Furthermore, using a PE Cluster Kit cBot-HS all the samples were clustered, and sequenced using an Illumina.

Fastp was used to remove the poly-N, and adapter reads from the raw data and process. The reads Q score over 50% was considered low quality, and Q score at 20 and 30 were identified as clean. Genome web browser National Center for Biotechnology Information/European Molecular Biology Laboratories-European Bioinformatics Institute (NCBI/Ensembl-EBI) was used as the reference genome with HISAT2 program (daehwankimlab.github.io/hisat2/manual/).

#### Integrated analysis of proteomics and transcriptomics statistics

The proteomic data from Progenesis and the transcript data were analyzed in R v4.1.2. Both proteomic intensities and transcript counts were normalised by Variance Stabilizing Normalisation (VSN).^174^ Due to the poor annotation status of Capra hircus gene, protein and pathway IDs, the data were 'humanised'; human protein-coding homolog gene IDs were assigned to matchable Goat IDs using the getLDS function in the biomaRt package.^175^ In total, 27552 IDs across the proteomic and transcriptomic datasets were mapped to 18658 human IDs. Further analysis was performed on the human IDs.

##### Removal of sample outliers

The outliers of AF and sham samples were identified by conducting principal component analysis on the individual and shared proteomics and transcriptomics data, then the outliers were removed to increase the confidence of the study (Please refer to the PCA plot showing the outliers of combined proteomics and transcriptomics data in the Figure S15).

For each AF vs. Control comparison, t-statistics were calculated for the AF versus control comparison for each gene. For all human Reactome pathways containing genes found in the dataset, we calculated rank-based 2D enrichment MANOVA *p*-values after^57^ for all pathways with at least 2 genes found on both sides of each paired comparison, over the three proteomics datasets and the transcriptomics dataset. Missing values for genes that had non-missing value at least one in any proteomics dataset were treated as ranked last; conversely, genes with no non-missing values except in the transcriptomics dataset were treated as missing (and excluded from ranking) in the proteomics datasets. To correct for multiple testing, pathway *p*-values were converted to q-values using the fdr tool package.^176^ We set a threshold of 1% FDR for significance.

#### Protein quantitation

Sample fractions EL or TL were mixed at a ratio of 1:1 with radio-immunoprecipitation (RIPA) buffer (Thermo Scientific). Protein concentrations of EL fractions and TL were determined using the Bicinchoninic acid assay (BCA Protein Assay Kit, Thermo Scientific). Bovine serum albumin was used as a protein standard, and serial dilutions were prepared from the initial stock concentration of 2 mg/mL to prepare a standard curve. To ensure accuracy and reproducibility, protein assays were performed in triplicate. Absorbance values were measured at 562 nm. Protein concentrations were calculated by linear regression analysis.^21^

#### SDS/PAGE gel preparation and Western blotting

Sample fractions EL and TL were solubilised, and proteins denatured using SDS/PAGE loading buffer (bio rad) and 2-mercaptoethanol (Sigma-Aldrich). Proteins were separated by gel electrophoresis (NW04120BOX, NuPAGE 4%–12% Bis-Tris protein gels, 20X MES buffer). The gel was transferred to nitrocellulose membrane (NC) (Bio-Rad) for protein transfer (X-cell-II blot module, Thermo Fisher Scientific). NC membrane was incubated in 5% skimmed milk. The primary antibodies anti-GYG1 (1:1000, sc-271109, Santa Cruz), anti-Rab11 (1:1000, ab65200, abcam) were incubated. Goat anti-rabbit antibody (1:2,500, Dako P0448) was used as the secondary antibody to detect the protein markers. The secondary antibodies were detected via chemiluminescence using Westar Supernova (XLS3, 0020, Cyanogen) and the protein bands were visualized in a ChemiDoc XRS + imager (Bio-rad with image Lab software).^21^ Un-paired t test was performed on the comparative band intensity values obtained from the Western blots to understand the significant changes of the protein levels between AF and sham/control goat groups.

##### Preparation of atrial tissue extracts for Westernblot analysis

Frozen left atrial regions were weighed and homogenised at a concentration of 20 mg wet weight/mL of RIPA buffer (10 mM Tris-HCl, pH 8, 150 mM NaCl, 0.5% IGEPAL-CA630, 0.5% sodium deoxy-cholate, 0.1% SDS) containing protease inhibitors (5 mM EDTA, 1 mM EGTA, 5 mg/mL leupeptin, 5 mg/mL aprotinin, 2 mg/mL pepstatin, 120 mg/mL Pefabloc, 2 mM 1,10-phenanthroline), using 10 strokes of a Dounce homogenizer connected to an overhead stirrer (Wheaton, NJ) set to speed 4. The resulting homogenate was then centrifuged at 10,000 x *g* at 4°C for 10 min, and the supernatant was collected. Total protein content of samples was estimated using a bicinchoninic acid protein assay kit (Pierce) and equalised to 1 mg/mL using RIPA buffer. Samples were frozen as 1 mL aliquots at −80°C or boiled for 3 min at 100°C in Laemmli tris-glycine sample buffer (62.5 mM Tris-HCl, pH 6.8, 10% v/v glycerol, 2% w/v SDS, 0.01% w/v bromophenol blue) and subsequently used for Western immunoblot.

##### SDS-PAGE and Westernblot analysis

14% polyacrylamide tris-glycine gels were used immunoblot detection of LC3-I and II (Cell Signaling Technology, UK), as well as phospho- and total p70S6 kinase (Cell Signaling Technology, UK). Tris-glycine gel solutions were made from a 30% T (total w/v %), 2.6% C (cross-linker w/v %) acrylamide/bisacrylamide stock solution (Sigma, UK) and run on a triple-wide gel system (C.B.S. Scientific, USA). 50 μg of protein from each sample was loaded onto gels and run at 150 V. Proteins were transferred onto Protran reinforced nitrocellulose membrane (0.2 μm pore size, Amersham, UK) at 400 mA for 2 h at 4°C. Uniform transfer of proteins onto nitrocellulose was confirmed by reversible staining with Ponceau S (0.1% w/v, 1% acetic acid, Sigma). Membranes were then blocked for 1 h at room temperature in 5% skimmed milk/TBS-T (Tris-buffered saline solution containing 0.1% Tween 20), then washed (three times for 5 min) in TBS-T, and incubated with appropriate primary antibodies in a 1% BSA/TBS-T solution for 2 h at room temperature or overnight at 4°C. Membranes were washed (three times for 5 min) in TBS-T before horseradish peroxidase-conjugated secondary antibodies (sheep anti-mouse HRP (Li-Cor Biosciences, UK) and donkey anti-rabbit (Li-Cor Biosciences, UK) made in 2.5% milk/TBS-T) were added for 1 h at room temperature. Membranes were washed (three times for 20 min) in TBS-T, and the proteins of interest were visualized using chemiluminescent substrates (Pierce). Fuji Super RX film (FujiFilm, Dusseldorf, Germany) band intensities were quantified using Image Studio Lite software (Li-Cor Bioscience, UK).

#### Lysosomal hydrolase activity assays

To fluorometrically measure the lysosome enzyme levels, artificial sugar substrates containing the fluorophore 4-methylumbelliferone (4-MU) were used. For measuring β-hexosaminidase activity, 3 mM 4-MU N-acetyl-β-D-glucosaminide (Sigma Aldrich) in 200 mM sodium citrate buffer, pH 4.5 and 0.1% Triton X-100 was used as substrate. For β-galactosidase activity, 1 mM 4-MU β-D-galactopyranoside (Sigma Aldrich) in 200 mM sodium acetate buffer, pH 4.3, 100 mM NaCl, and 0.1% Triton X-100 was used as substrate. The reaction was stopped by adding chilled 0.5 M Na2CO3, and the released fluorescent 4-MU was measured in a Clariostar OPTIMA plate reader (BMG Labtech, Ortenberg, Germany) with an excitation at 360 nm and emission at 460 nm. A standard curve for free 4-MU was used to calculate the enzyme activity. Results were calculated as total Units of enzyme activity (nmol/h) and normalised with respect to protein content.^21^

#### Sample preparation for electron microscopy (EM)

Left atrial samples (from *N* = 3 sham animals and 3 AF animals) were prepared by chemical fixation. Approximately 1 mm^3^ pieces of tissue from the left atria were rapidly dissected and fixed in Karnovsky fixative containing paraformaldehyde 4%, glutaraldehyde 5%, cacodylate buffer 80 mM, pH 7.4,^177^ and were embedded in Spurr’s resin.^178^ Sections were cut to approximately 70–80 nm thickness (Reichart Ultracut) then post-stained with 2% aqueous uranyl acetate and Reynolds lead citrate for contrast. Images were obtained using a transmission electron microscope (Thermo Fisher Tecnai T12 TEM, operated at 120 kV, using a Gatan OneView camera). Extensive quantitative ultra-structure studies have been published on the goat AF model. We performed unbiased qualitative EM analysis to cross check our results with that of the goat AF studies performed by Ausma et al.^39^

##### Manual glycogen EM image analysis

Analysis and measurements were made using Gatan DigitalMicrograph and ImageJ software. Raw EM images were analyzed using ImageJ (Version 2.3.0/1.53s). The area of interest was manually selected using the box tool, avoiding cellular organelles such as Golgi, lysosome, mitochondria etc. 5–7 boxes per image were selected in order to cover most of the cytosolic space. Box sizes in pixels were generated by ImageJ. Glycogen quantification was evaluated manually using the multi-point tool, one point represents one glycogen. The overall number of points was recorded as an output by ImageJ. The results are presented as glycogen counts per nmˆ2.

##### Automated glycogen EM image analysis

We performed independent automated image analysis to cross-check the manual counts performed. This removes user bias. Images were processed to obtain glycogen count estimates from pre-selected regions of interest. The image processing pipeline begins by cropping out each area of interest from the larger image. Noise in the cropped image is then reduced by applying a Gaussian filter to smooth the image and a min filter to accentuate dark areas. Following this, the image is binarized at a color threshold that separates the dark glycogen shapes from the background. Foreground shapes are extracted from the binary image and counted if their area is larger than a minimum size. For larger foreground shapes which contain multiple glycogen, the count is estimated as the maximum number of glycogen deposits which can fit into the area. The parameters for filtering foreground shapes by size and the threshold for binarizing images were selected by visually inspecting results from the image processing pipeline. All image analysis was conducted in Python 3.10.11 with opencv-python 4.7.0 (source code available at https://doi.org/10.5281/ZENODO.7892313).

### Additional resources

All the required resources are provided in the STAR methods and supplementary data.

## Supplemental information

Document S1. Figures S1‒S18 and Tables S2.2–S3.1

Table S1.1. The most significantly regulated gene list obtained from transcriptomics analysis

Table S1.2. The most significantly regulated protein list obtained from TL fraction of proteomics analysis

Table S2.1. The list of pathways from the integrated analysis of EL, TL proteomics and transcriptomics integrated analysis

Table S3.1. The list of most significantly regulated Reactome pathways identified from the EL proteomics entity input

Table S4. Progenesis data endolysosome fraction

Table S5. Progenesis data tissue lysate fraction
