# Supplementary material for: Compartmentalization proteomics revealed endolysosomal protein network changes in a goat model of atrial fibrillation
Source: iScience. 2024 Mar 28;27(6):109609. doi: 10.1016/j.isci.2024.109609 (PMC11141153; doi:10.1016/j.isci.2024.109609)
Supplement: Document S1. Figures S1‒S18 and Tables S2.2–S3.1 [file mmc1.pdf]

iScience, Volume *27*

Supplemental information

Compartmentalization proteomics revealed endolysosomal protein network changes in a goat model of atrial fibrillation

Thamali Ayagama, Philip D. Charles, Samuel J. Bose, Barry Boland, David A. Priestman, Daniel Aston, Georgina Berridge, Roman Fischer, Adam P. Cribbs, Qianqian Song, Gary R. Mirams, Kwabena Amponsah, Lisa Heather, Antony Galione, Neil Herring, Holger Kramer, Rebecca A. Capel, Frances M. Platt, Ulrich Schotten, Sander Verheule, and Rebecca A. B. Burton

Supplemental Figures and Tables

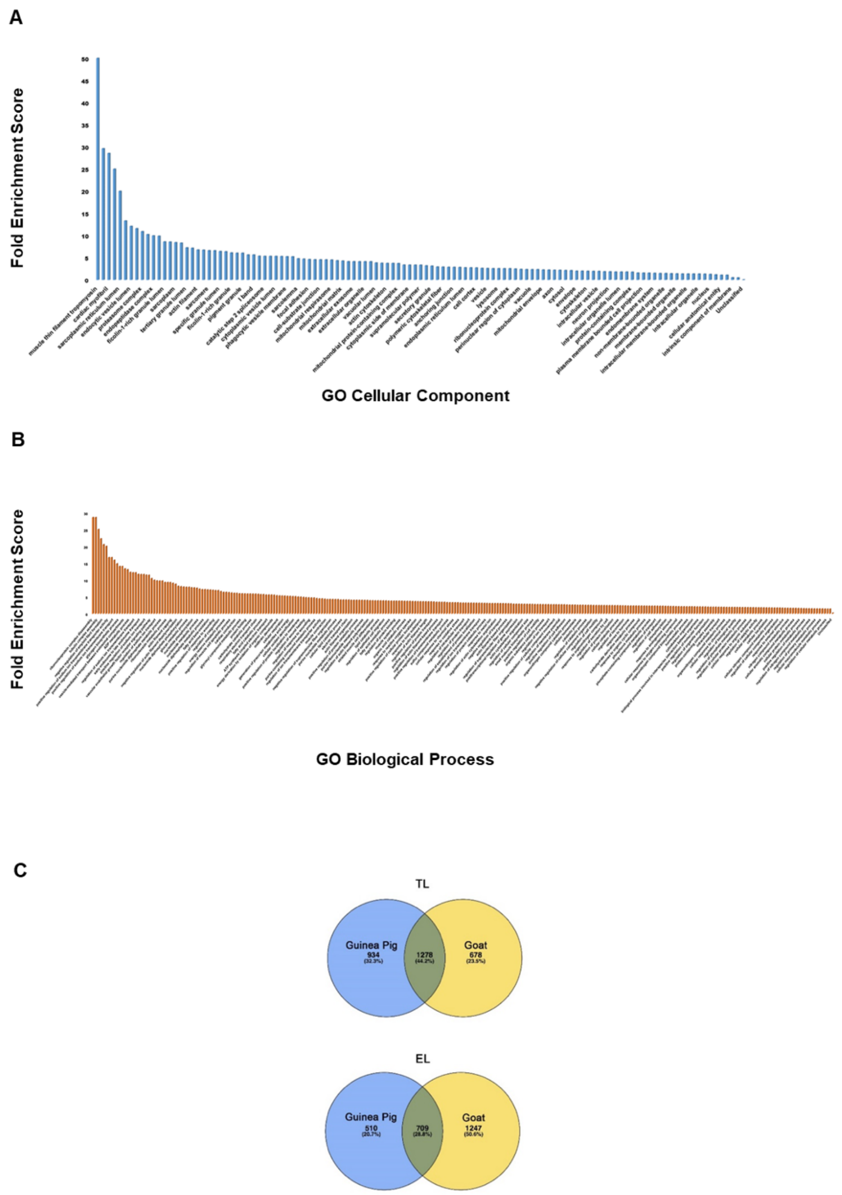

Figure S1: The enrichment of regulated proteins in AF goat model compared to the total human proteome **relating to Figure 1 and 3. A) and B)** The over representation test was performed using the converted C. *hircus* protein identifiers to H. *sapiens*. The higher fold enrichment scores were plotted against the Gene Ontology functional and anatomical parameters, Cellular component and Biological process. **C)** Species comparison between C. *porcellus* and C. *hircus* TL and EL fractions against the protein yield discovered from Ayagama *et al.* 2021 study [21] to understand the protein/gene recovery.

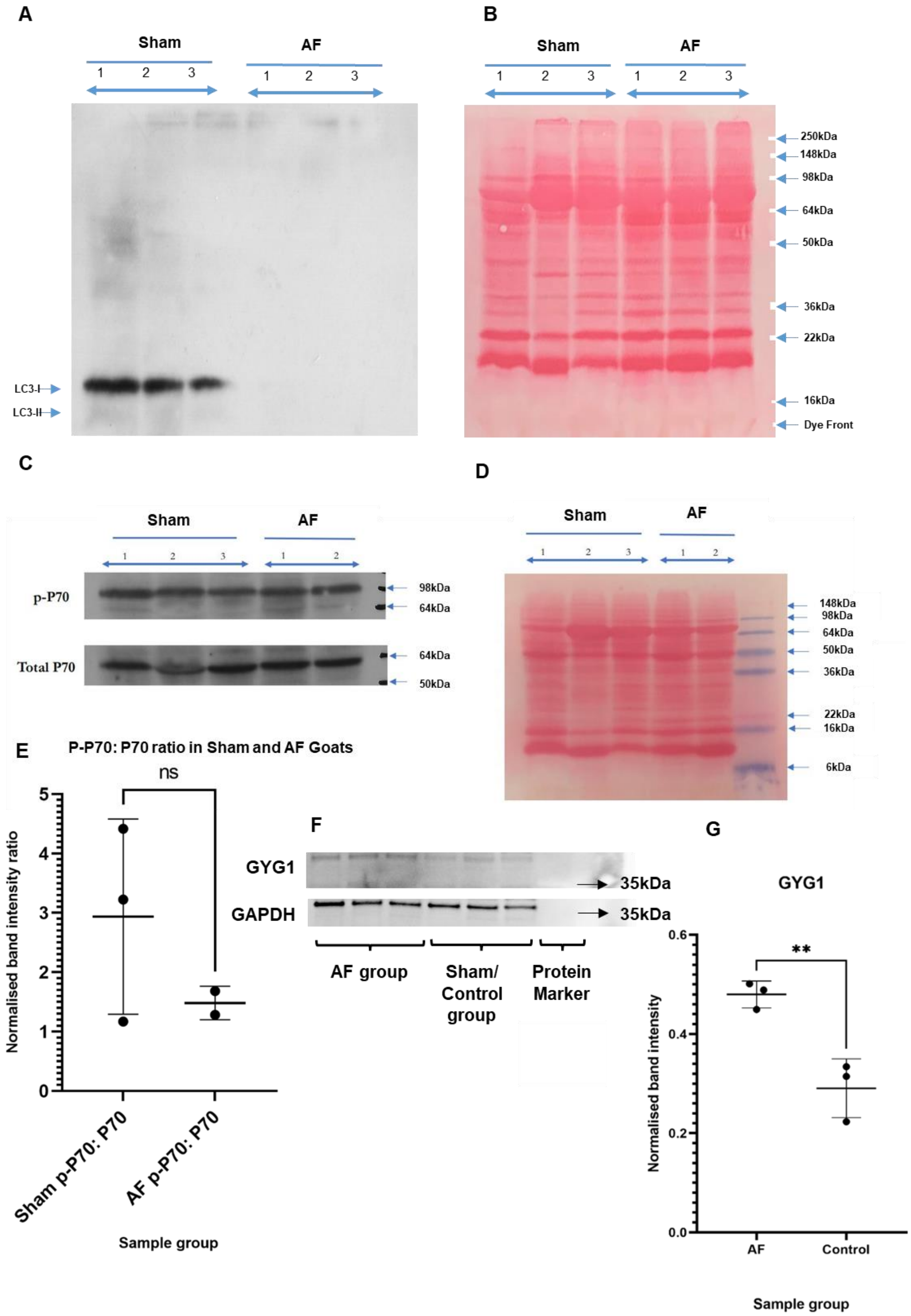

Figure S2: Western blotting for protein identification and quantification relating to Figure 1, 2 and 3. A) and B) The absence of the LC3- I and LC3-II proteins showed an overactive autophagic flux in the LA tissue of the AF goat model, and a Ponceau blot of the LC3-I and LC3-

II membrane was prepared to observe equal protein loading. **C), D) and E)** Western blotting of the mTOR-specific epitope p-P70 and total P70 levels. The p-P70 protein level change is insignificant compared to total P70. **F) and G)** Western Blotting of Glycogenin-1 and GAPDH. GYG1 western blots displayed a significant upregulation of GYG-1 protein in the LA tissue of the AF goat model ($p = 0.007$).

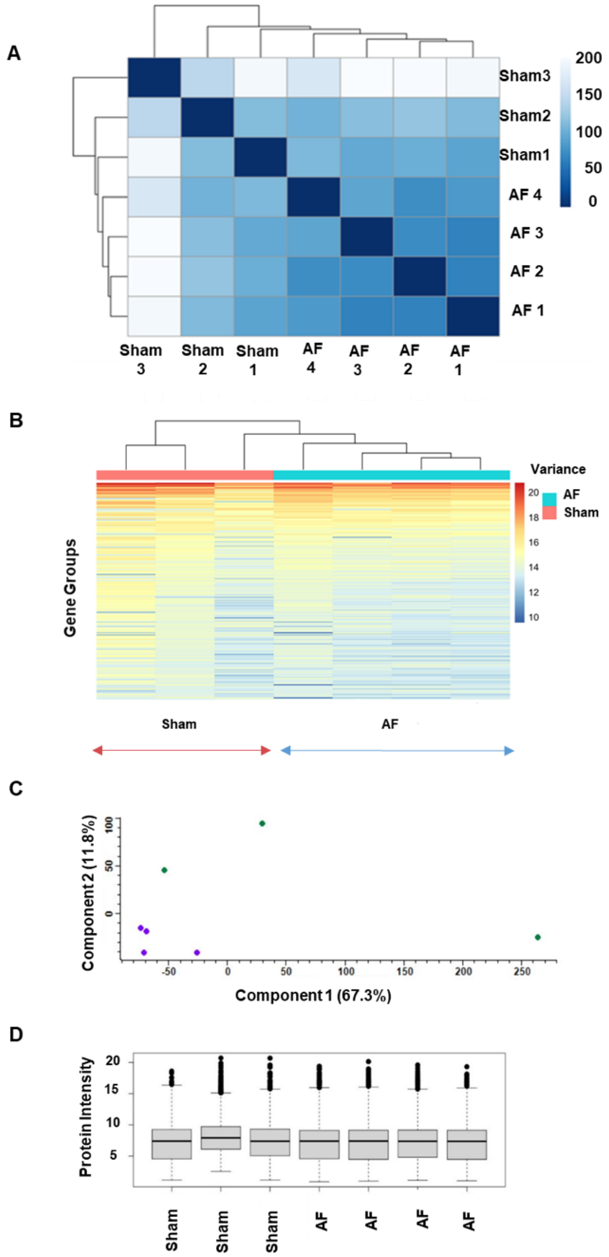

Figure S3: Quality control analysis for transcriptomics relating to Figure 4A. A) Distance matrix heatmap to determine the distance between the AF and sham samples according to their gene groups. **B)** A heat map of the transcripts obtained from all the samples after analysed for hierarchical clustering. **C)** PCA plot to verify the sample distribution between AF (purple circles) and sham (green circles) groups. Component 1 showed a deviation of 67.3% between the groups and component 2 showed a 11.8% of deviation between the samples of sham group biological replicates. **D)** Whisker plot showing the transcript distribution between the AF and sham biological replicates.

A

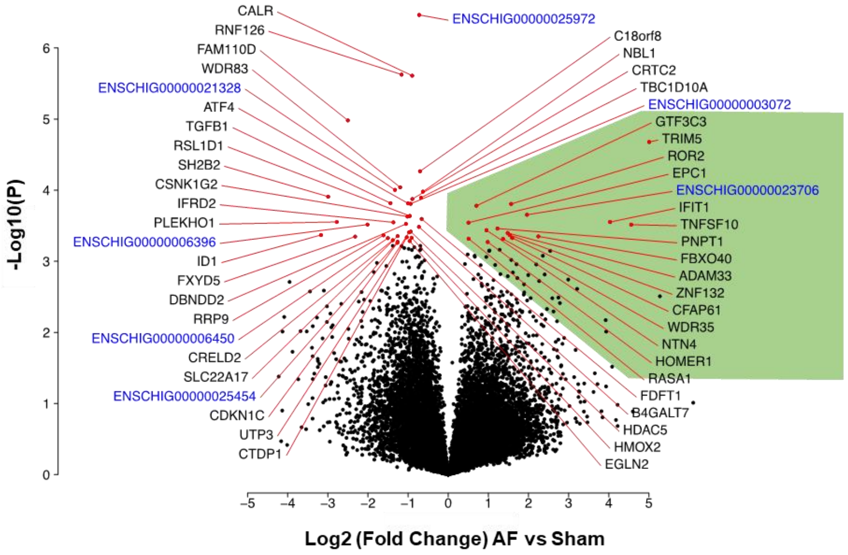

B

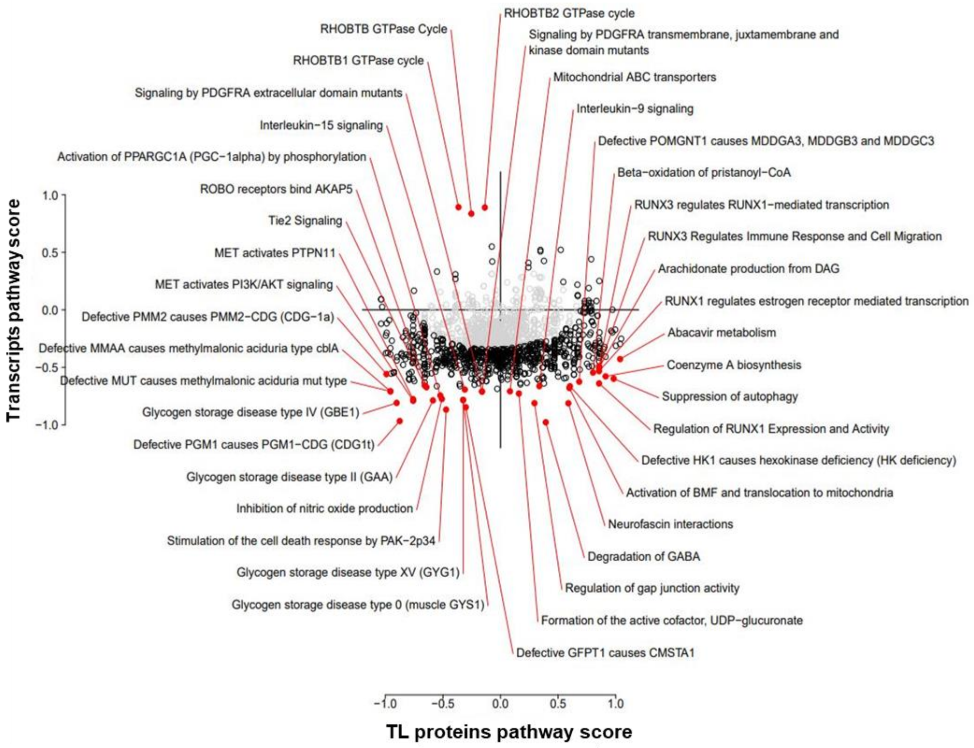

Figure S4: Transcriptomics and integrated analysis (TL) relating to Figure 1(B). A) Volcano plot of the most significantly regulated genes identified from the transcriptomics analysis**. B) Integrated Analysis;** TL protein pathways vs mRNA transcript pathway scores. The pathways

falling well under the horizontal line are up/downregulated more strongly in the proteomics than the transcriptomics vice versa for pathways falling above the line. grey = nonsignificant pathways, black= significant pathways red= highest-significant pathways (FDR=0.01) and the black horizontal line indicates y=x.

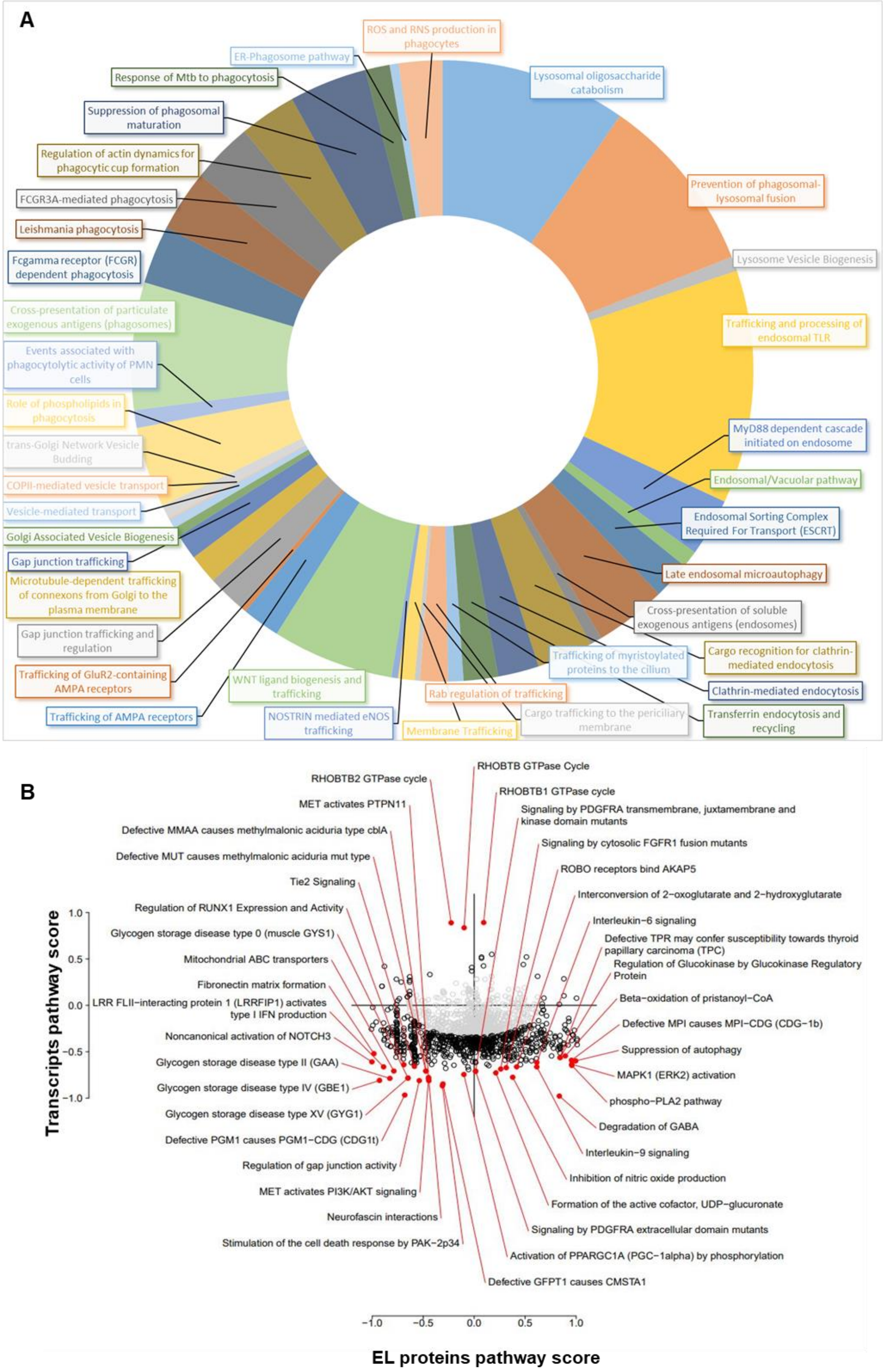
A
ROS and RNS production in phagocytes
ER-Phagosome pathway
Response of Mtb to phagocytosis
Suppression of phagosomal maturation
Regulation of actin dynamics for phagocytic cup formation
FCGR3A-mediated phagocytosis
Leishmania phagocytosis
Fcgamma receptor (FCGR) dependent phagocytosis
Cross-presentation of particulate exogenous antigens (phagosomes)
Events associated with phagocytolytic activity of PMN cells
Role of phospholipids in phagocytosis
trans-Golgi Network Vesicle Budding
COPII-mediated vesicle transport
Vesicle-mediated transport
Golgi Associated Vesicle Biogenesis
Gap junction trafficking
Microtubule-dependent trafficking of connexons from Golgi to the plasma membrane
Gap junction trafficking and regulation
Trafficking of GluR2-containing AMPA receptors
Trafficking of AMPA receptors
WNT ligand biogenesis and trafficking
NOSTRIN mediated eNOS trafficking
Membrane Trafficking
Rab regulation of trafficking
Cargo trafficking to the periciliary membrane
Transferrin endocytosis and recycling
Clathrin-mediated endocytosis
Trafficking of myristoylated proteins to the cilium
Cargo recognition for clathrin-mediated endocytosis
Cross-presentation of soluble exogenous antigens (endosomes)
Late endosomal microautophagy
Endosomal Sorting Complex Required For Transport (ESCRT)
Endosomal/Vacuolar pathway
MyD88 dependent cascade initiated on endosome
Trafficking and processing of endosomal TLR
Lysosome Vesicle Biogenesis
Prevention of phagosomal-lysosomal fusion
Lysosomal oligosaccharide catabolism
B
RHOBTB2 GTPase cycle
RHOBTB GTPase Cycle
RHOBTB1 GTPase cycle
MET activates PTPN11
Signaling by PDGFRA transmembrane, juxtamembrane and kinase domain mutants
Defective MMAA causes methylmalonic aciduria type cblA
Signaling by cytosolic FGFR1 fusion mutants
Defective MUT causes methylmalonic aciduria mut type
ROBO receptors bind AKAP5
Tie2 Signaling
Interconversion of 2−oxoglutarate and 2−hydroxyglutarate
Regulation of RUNX1 Expression and Activity
Interleukin−6 signaling
Glycogen storage disease type 0 (muscle GYS1)
Defective TPR may confer susceptibility towards thyroid papillary carcinoma (TPC)
Mitochondrial ABC transporters
Regulation of Glucokinase by Glucokinase Regulatory Protein
Fibronectin matrix formation
Beta−oxidation of pristanoyl−CoA
LRR FLII−interacting protein 1 (LRRFIP1) activates type I IFN production
Defective MPI causes MPI−CDG (CDG−1b)
Noncanonical activation of NOTCH3
Suppression of autophagy
Glycogen storage disease type II (GAA)
MAPK1 (ERK2) activation
Glycogen storage disease type IV (GBE1)
phospho−PLA2 pathway
Glycogen storage disease type XV (GYG1)
Degradation of GABA
Defective PGM1 causes PGM1−CDG (CDG1t)
Interleukin−9 signaling
Regulation of gap junction activity
Inhibition of nitric oxide production
MET activates PI3K/AKT signaling
Formation of the active cofactor, UDP−glucuronate
Neurofascin interactions
Signaling by PDGFRA extracellular domain mutants
Stimulation of the cell death response by PAK−2p34
Activation of PPARGC1A (PGC−1alpha) by phosphorylation
Defective GFPT1 causes CMSTA1
Transcripts pathway score
1.0
0.5
0.0
−0.5
−1.0
−1.0
−0.5
0.0
0.5
1.0
EL proteins pathway score

Figure S5: Pathways representing the acidic organelles and integrated analysis (EL) relating to Figure 1(A). A) Pathways representing the acidic organelles, vesicles and trafficking network from integrated analysis are represented using a donut chart against the enrichment scores. Data used to create this plot can be found in Supplementary Table S2.1. **B)** Integrated analysis of EL protein pathways vs mRNA transcript pathway scores. Pathways represented in black and red are significantly altered (based on null hypothsis of no change) in either the transcriptomic data, proteomic data, or both. Pathways in the lower left and upper right (i.e. close to y=x) are regulated in the same direction in both modalities. Pathways in the upper left and lower right (i.e. close to y=-x) are regulated in the opposite direction in the two modalities. Grey = nonsignificant pathways; Black = significantly altered pathways (FDR<0.01); Red = top 40 most significant pathways.

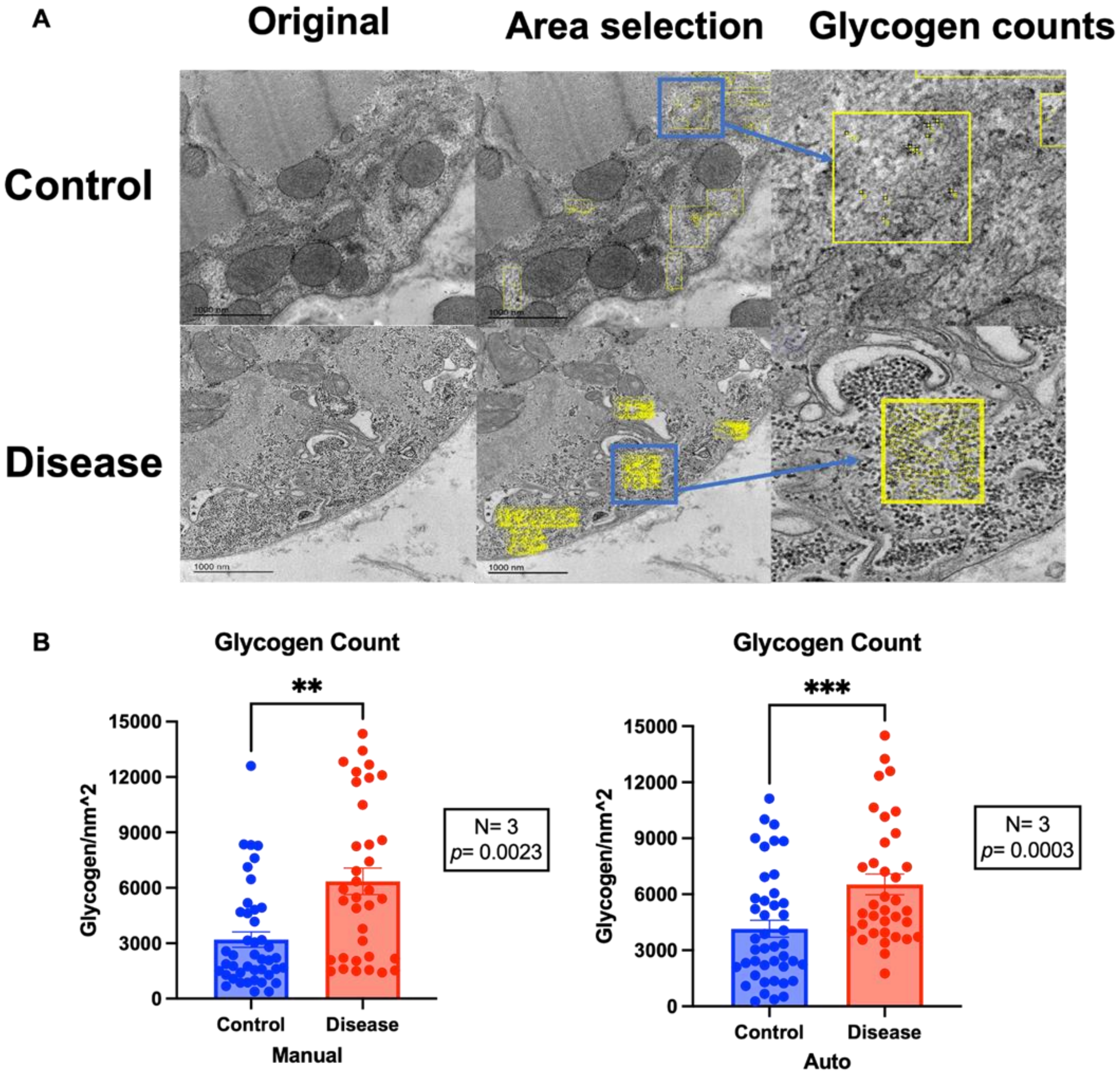

Figure S6: Electron microscopy analysis of glycogen expression in control vs AF goat atrial tissue relating to Figure 5. A) Demonstration of cardiac myocyte under EM. Cytosolic area was selected manually (blue box), then the glycogen (black spot, labelled yellow) was counted both manually and automatically (user independent). **B)** Both manual and automated glycogen quantification statistically reveal the same result, that glycogen count was significantly higher in the disease samples. Manual count, disease mean glycogen count 6354±714.8/nm^2 vs. control mean glycogen count 3195±417.9/nm^2, p= 0.0023; Auto count disease mean glycogen count 6529±555.0/nm^2 vs. control mean glycogen count 4154±415.8/nm^2, p= 0.0003. N=3 for each group.

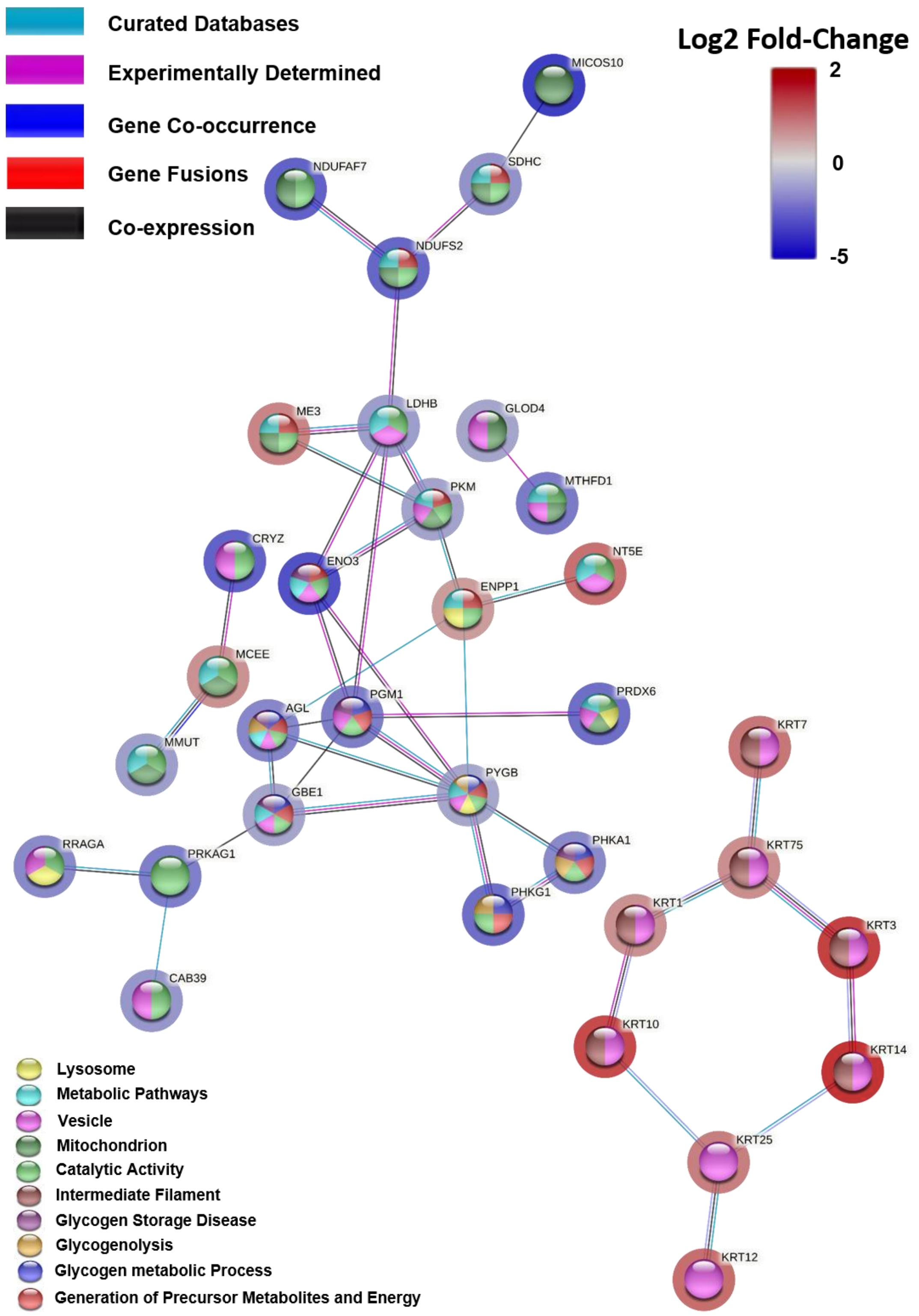

Figure S7: Association network in STRING. Tissue lysate (TL) Network Cluster 5 relating to Figure 1B. The most significant proteins that have a functional role of regulatory and catalytic activity are clustered together. Edges based on curated databases, experiments, gene co-

occurrences, gene fusions, co-expressions and the nodes are coloured according to their functional enrichments. The halo around the nodes displays the significant log2 fold-change.

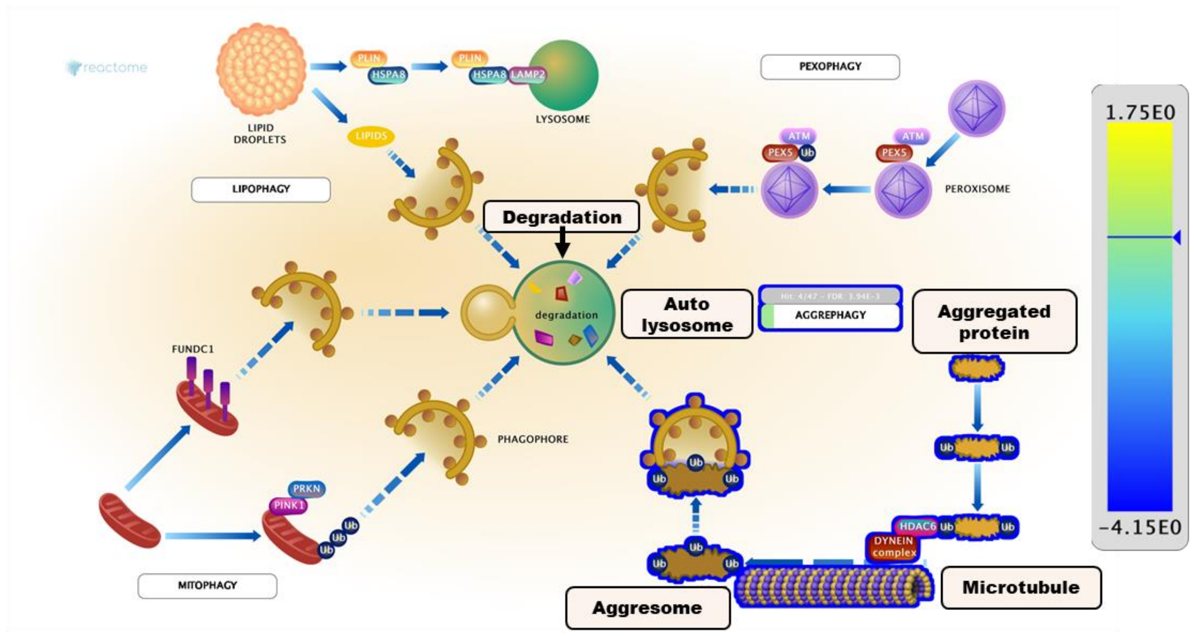

Figure S8: Cartoon of Aggresome formation relating to Figure 1. The majority of the misfolded protein degradation was detected by then over expressed ubiquitin levels in the left atrial cells of the AF goat model. (Colour code= yellow to blue gradient; yellow represent highest up regulation while blue represent highest down regulation. Grey box above the aggrephagy label indicates the pathway entities detected in the query dataset, the total number of entities in the pathway, and the FDR corrected probability score. The protein complexes with complete or part-coloured representation showed the total molecules present from the query data set to complete the complex. Arrow = Protein, protein complex name or protein function). Figure created using www.reactome.com pathway analysis.

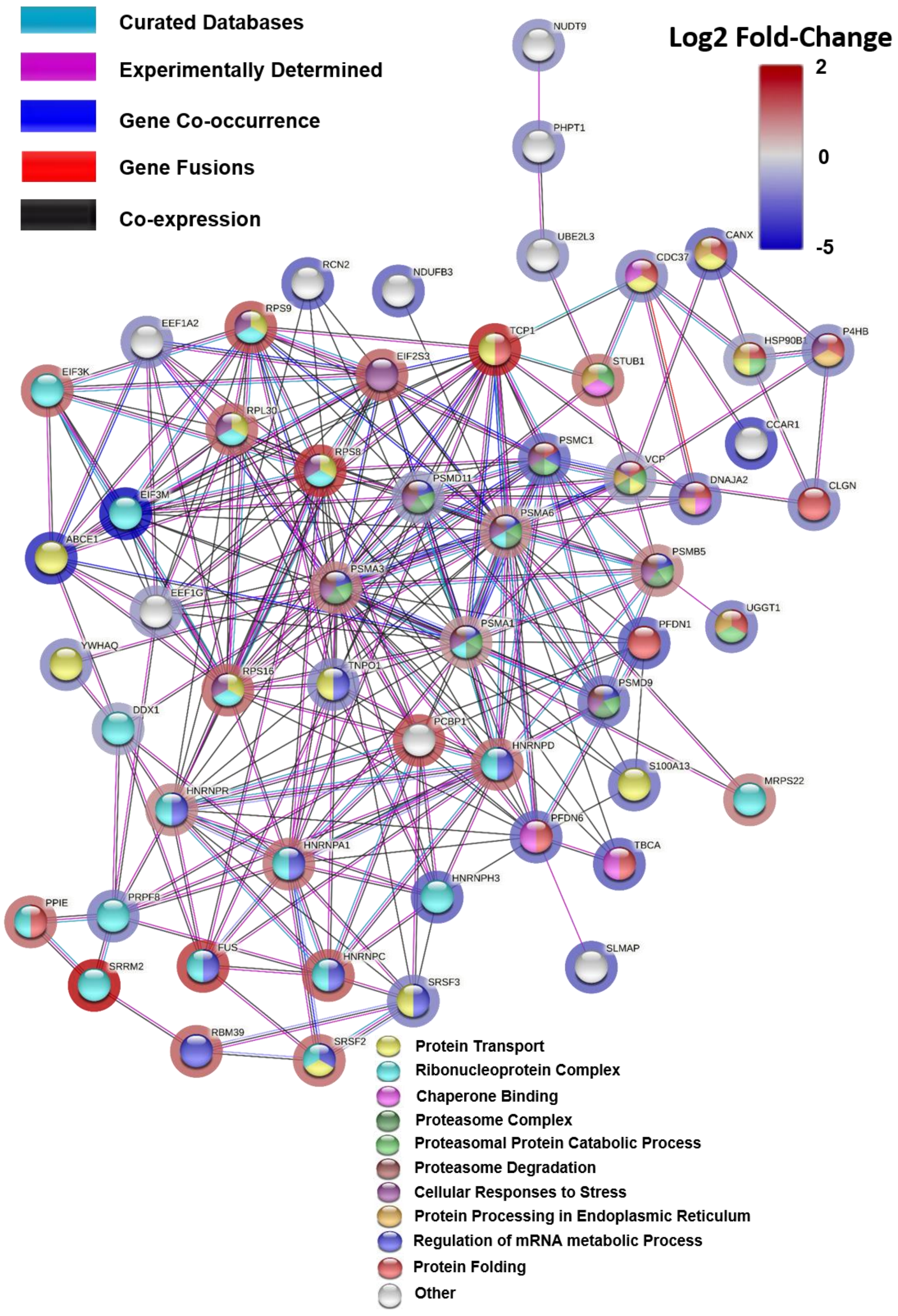
Curated Databases
Experimentally Determined
Gene Co-occurrence
Gene Fusions
Co-expression
Log2 Fold-Change
2
0
-5
NUDT9
PHPT1
UBE2L3
CANX
CDC37
RCN2
NDUFB3
RPS9
EEF1A2
TCP1
EIF2S3
STUB1
HSP90B1
P4HB
EIF3K
RPL30
CCAR1
RPS8
PSMC1
VCP
PSMD11
DNAJA2
CLGN
EIF3M
PSMA6
ABCE1
PSMB5
PSMA3
EEF1G
UGGT1
PSMA1
PFDN1
YWHAQ
RPS16
TNPO1
PSMD9
DDX1
PCBP1
HNRNPD
S100A13
MRPS22
HNRNPR
PFDN6
TBCA
HNRNPA1
HNRNPH3
PPIE
PRPF8
SLMAP
FUS
SRRM2
HNRNPC
SRSF3
RBM39
SRSF2
Protein Transport
Ribonucleoprotein Complex
Chaperone Binding
Proteasome Complex
Proteasomal Protein Catabolic Process
Proteasome Degradation
Cellular Responses to Stress
Protein Processing in Endoplasmic Reticulum
Regulation of mRNA metabolic Process
Protein Folding
Other

Figure S9: Association network in STRING. Tissue lysate (TL) Network cluster 3 relating to Figure 1B. The most significant proteins that were regulated as part of stress induced stimuli are clustered together and Edges based on curated databases, experiments, gene co-occurrences, gene fusions, co-expressions and the nodes are coloured according to their functional enrichments. The halo around the nodes displays the significant log2 fold-change.

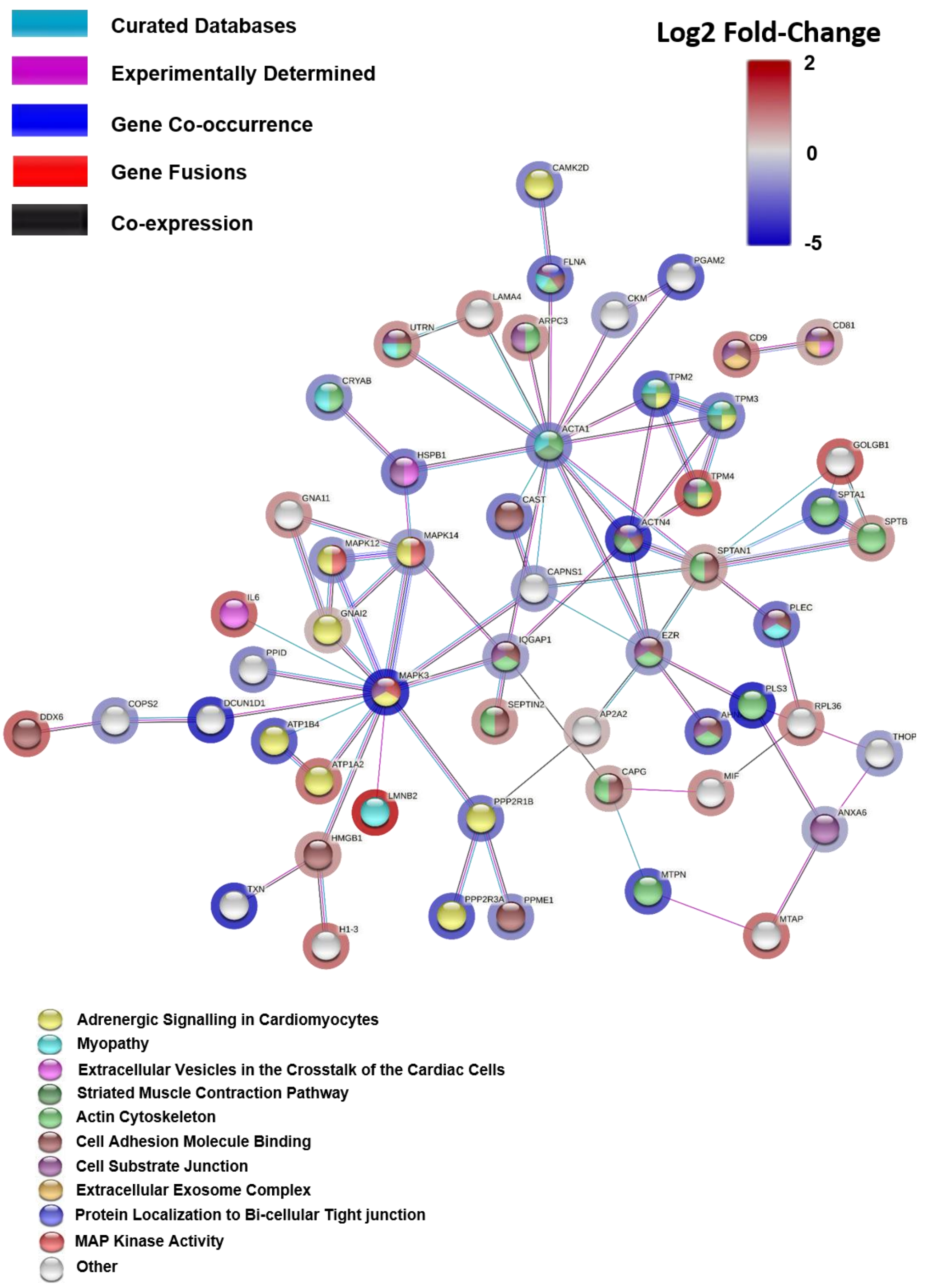

Figure S10: Association network in STRING. Tissue lysate (TL) Network cluster 1 relating to Figure 1B. The most significant proteins that represented dysfunctional atrial conduction are clustered together and Edges based on curated databases, experiments, gene co-occurrences, gene fusions, co-expressions and the nodes are coloured according to their functional enrichments. The halo around the nodes displays the significant log2 fold-change.

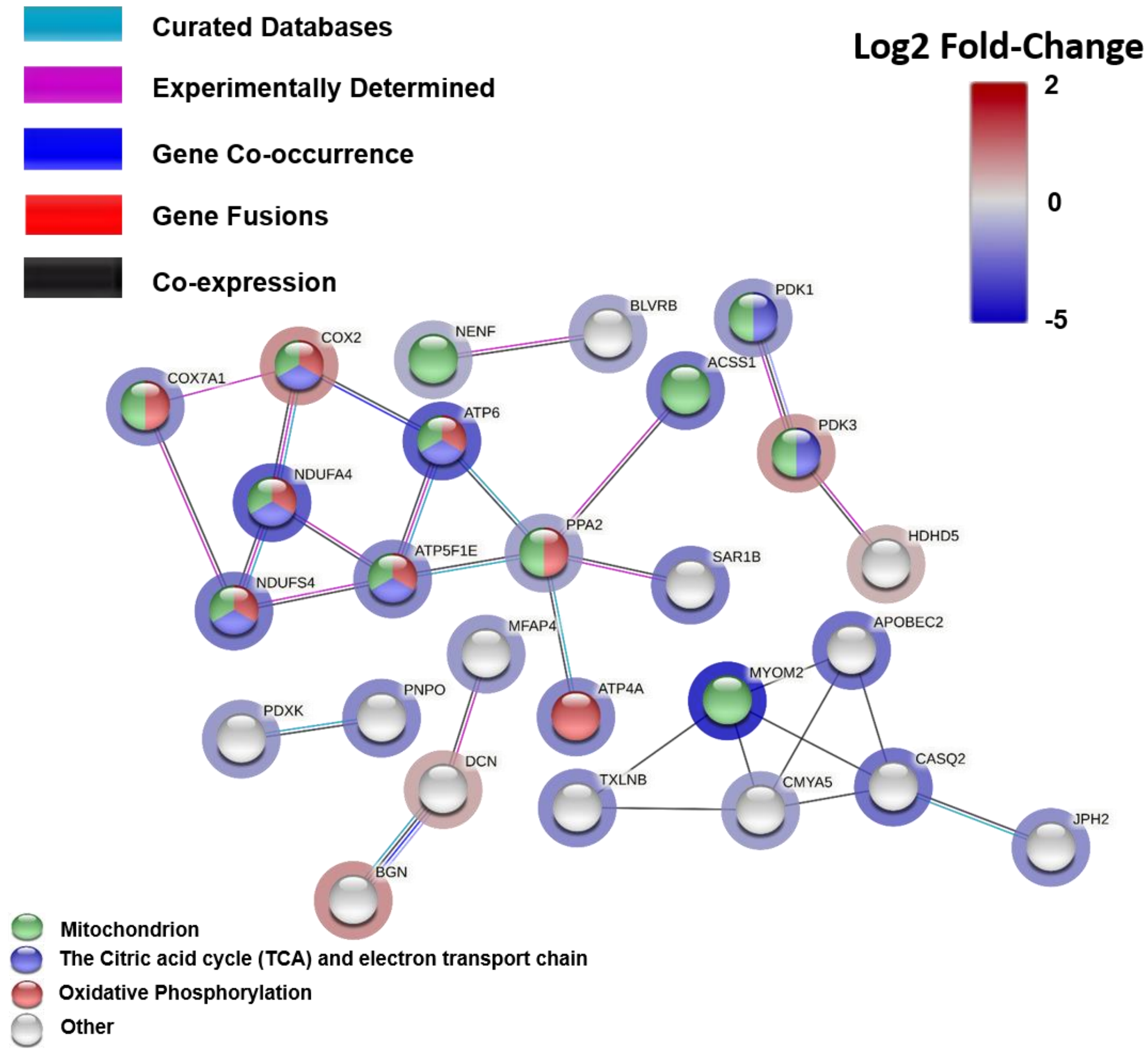

Figure S11: Association network in STRING. Tissue lysate (TL) Network Cluster 4 relating to Figure 1B. The most significant proteins that belong to the metabolism of mitochondria are clustered together and Edges based on curated databases, experiments, gene co-occurrences, gene fusions, co-expressions and the nodes are coloured according to their functional enrichments. The halo around the nodes displays the significant log2 fold-change.

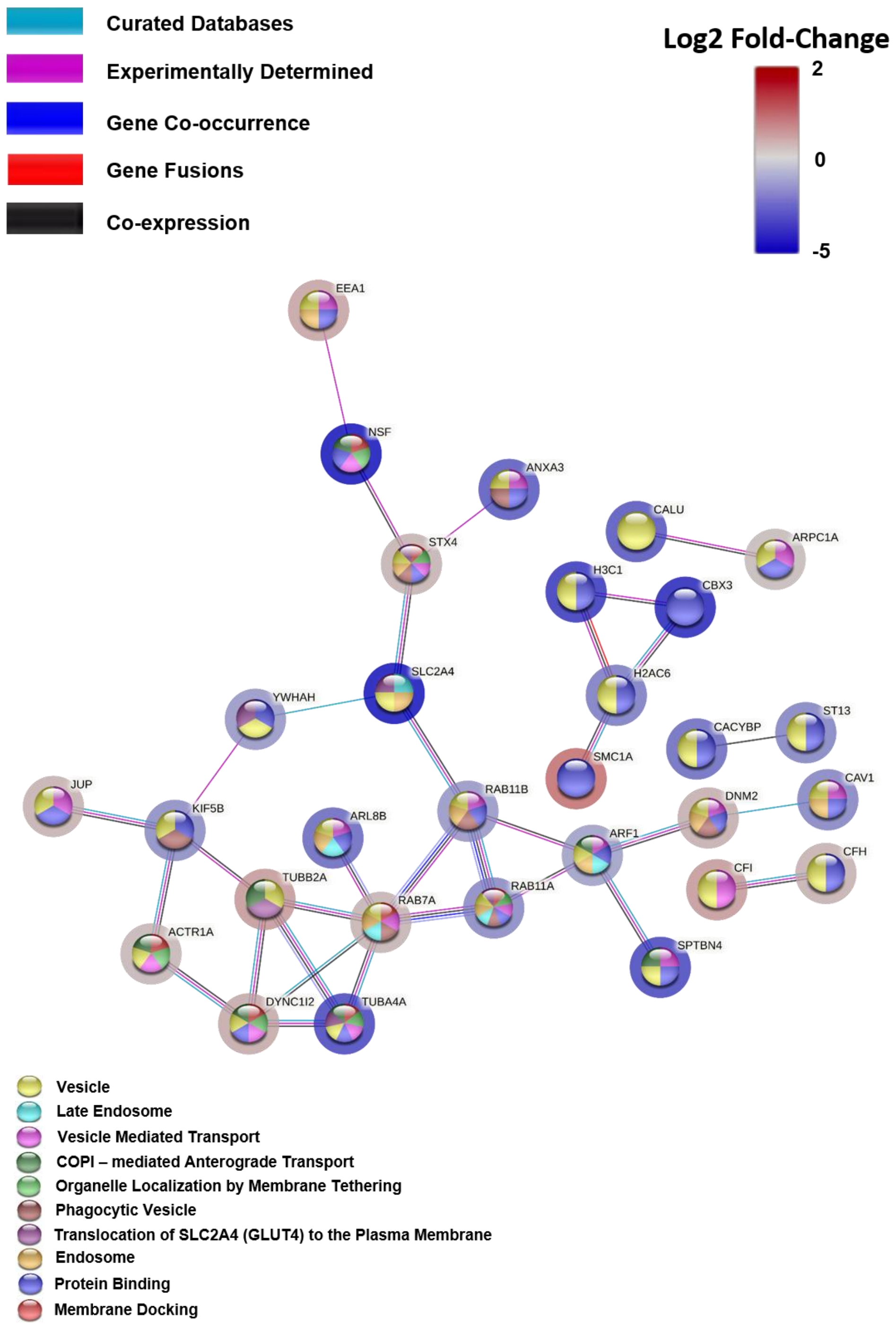

Figure S12: Association network in STRING. Tissue lysate (TL) Network Cluster 6 relating to Figure 1B. The most significant proteins that have a functional role of trafficking and transport are clustered together and the Edges based on curated databases, experiments, gene co-occurrences, gene fusions, co-expressions and the nodes are coloured according to their functional enrichments. The halo around the nodes displays the significant log2 fold-change.

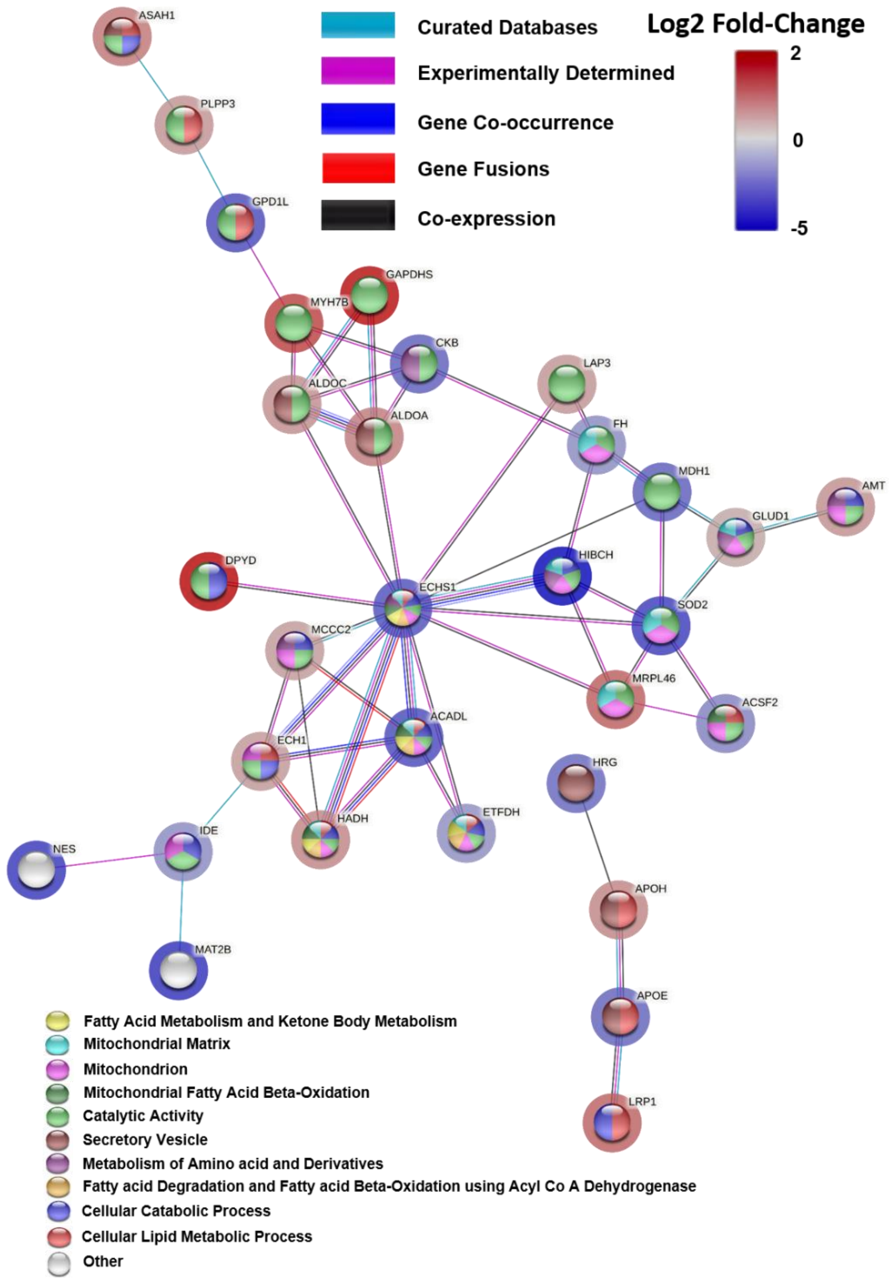

Figure S13: Association network in STRING. Tissue lysate (TL) Network cluster 2 relating to Figure 1B. The most significant proteins that represented cellular metabolisms are clustered together and Edges based on curated databases, experiments, gene co-occurrences, gene fusions, co-expressions and the nodes are coloured according to their functional enrichments. The halo around the nodes displays the significant log2 fold-change.

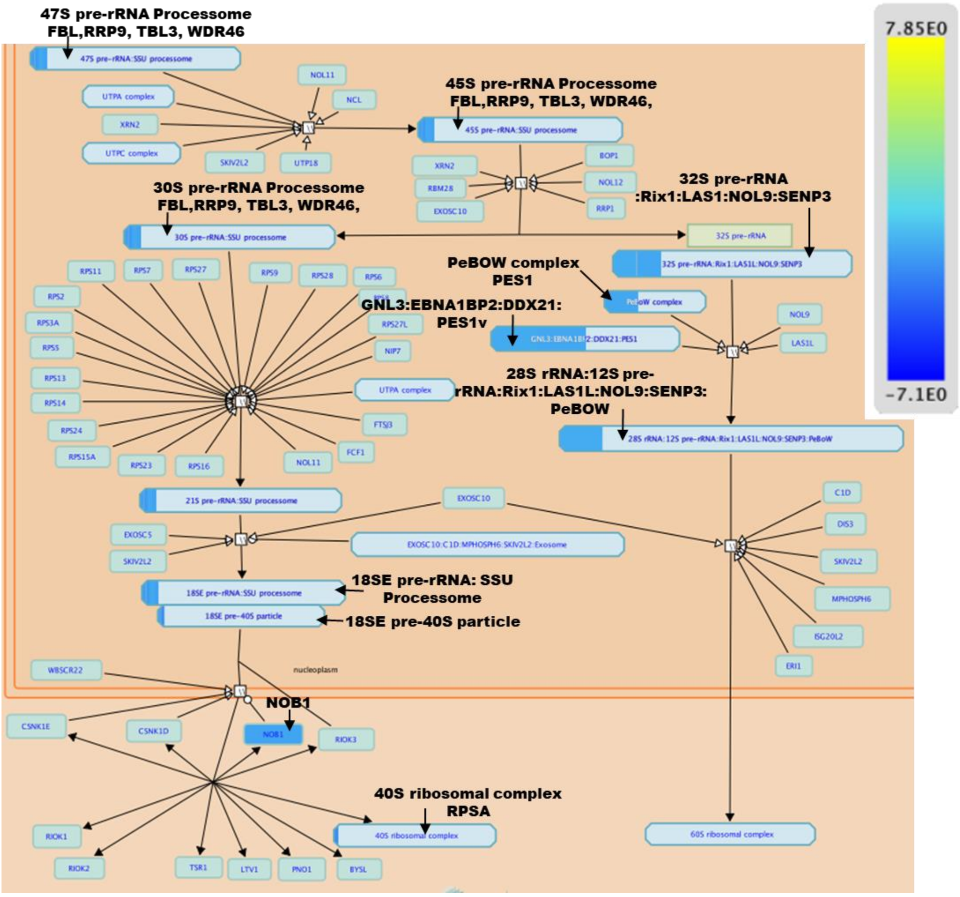

Figure S14: Transcriptomics analysis relating to Figure 1B. Transcriptomics analysis showing the Down regulation of the rRNA processing pathway in the nucleolus and cytosol of AF goat model. (Arrow = Gene name, protein complex name or protein function. Yellow = up regulation (log2 fold-change) and Blue= down regulation (log2 fold-change)). Pathway analysed using Reactome database.

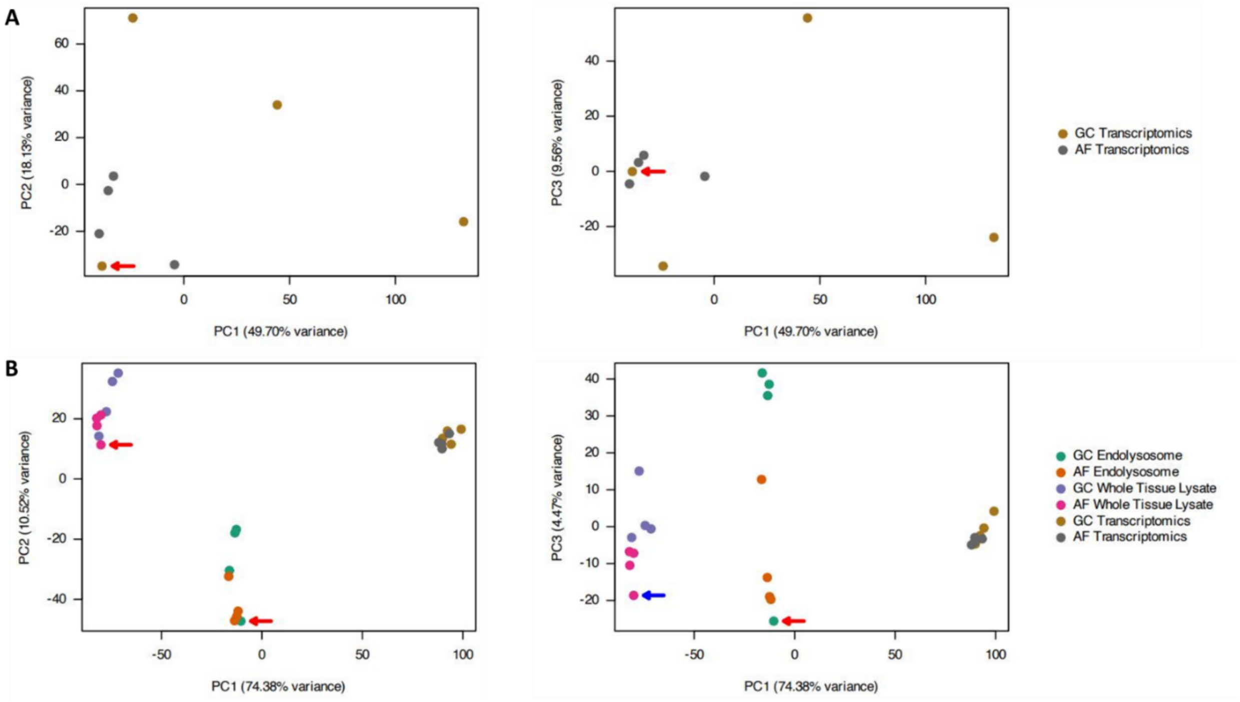

Figure S15: Biological replicate outlier analysis of transcriptomics-only and combined transcriptomics and proteomics data relating to STAR methods, removal of sample outliers. **A.** Principal Components Analysis plot for the transcriptomics-only data. Left panel - Principal Component (PC) 1 vs PC2. Right panel - PC1 vs PC3. Sample 38658 (indicated by red arrow), nominally a Goat Control (GC)/sham model sample, was observed to cluster with the Atrial Fibrillation (AF) samples. Further investigation suggested the possibility of sample contamination, so this sample was removed from the analysis. **B.** Principal Components Analysis plots for Whole Tissue Lysate and Endolysosome fraction sample proteomic data as well as the transcriptomic data after cross-modality integration. Left panel - Principal Component (PC) 1 vs PC2. Right panel - PC1 vs PC3. For consistency with the transcriptomics data processing, we applied similar strict criteria to outlier consideration in the proteomics dataset. Over all principal components, we identified endolysosome fraction GC sample 47220 (indicated by red arrow) and whole tissue lysate AF sample 45158 (indicated by blue arrow) as potential outliers, so we removed these samples from the analysis as well.

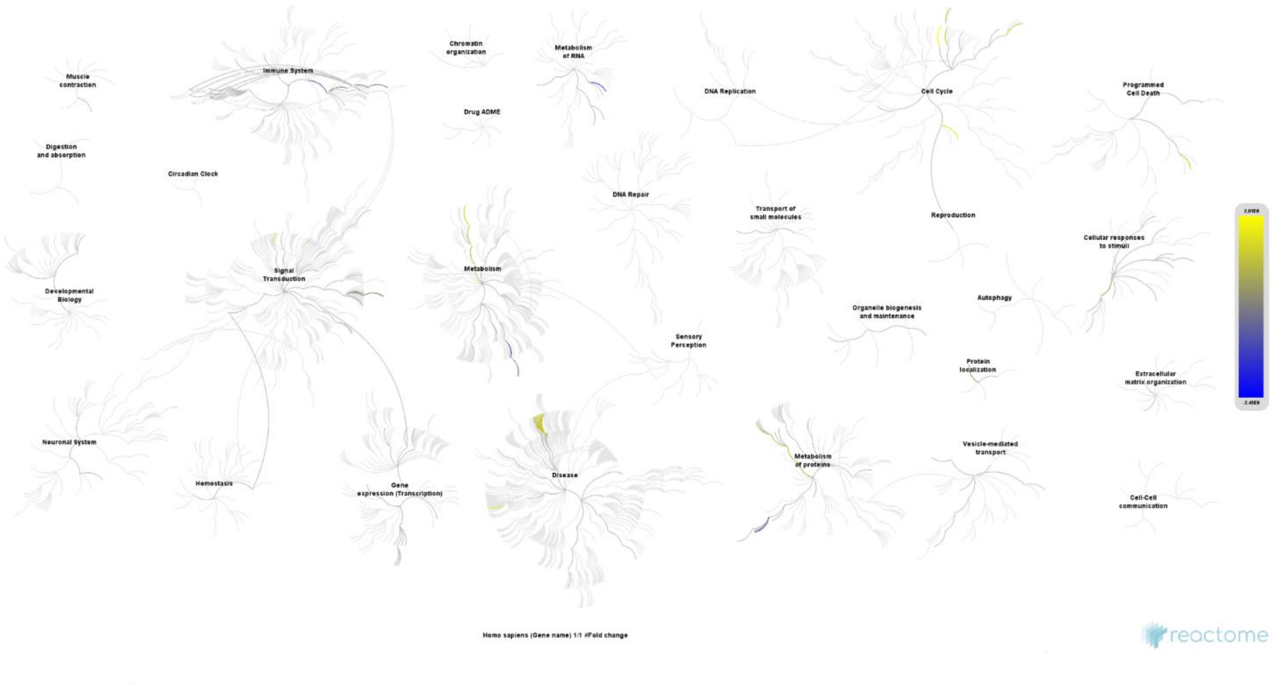

Figure S16: EL most significant proteins displayed in a Reactome genome-wide, hierarchical visualization of pathways in a space filling graph relating to Figure 1A. Highest up and down regulated pathways are depicted in yellow and blue.

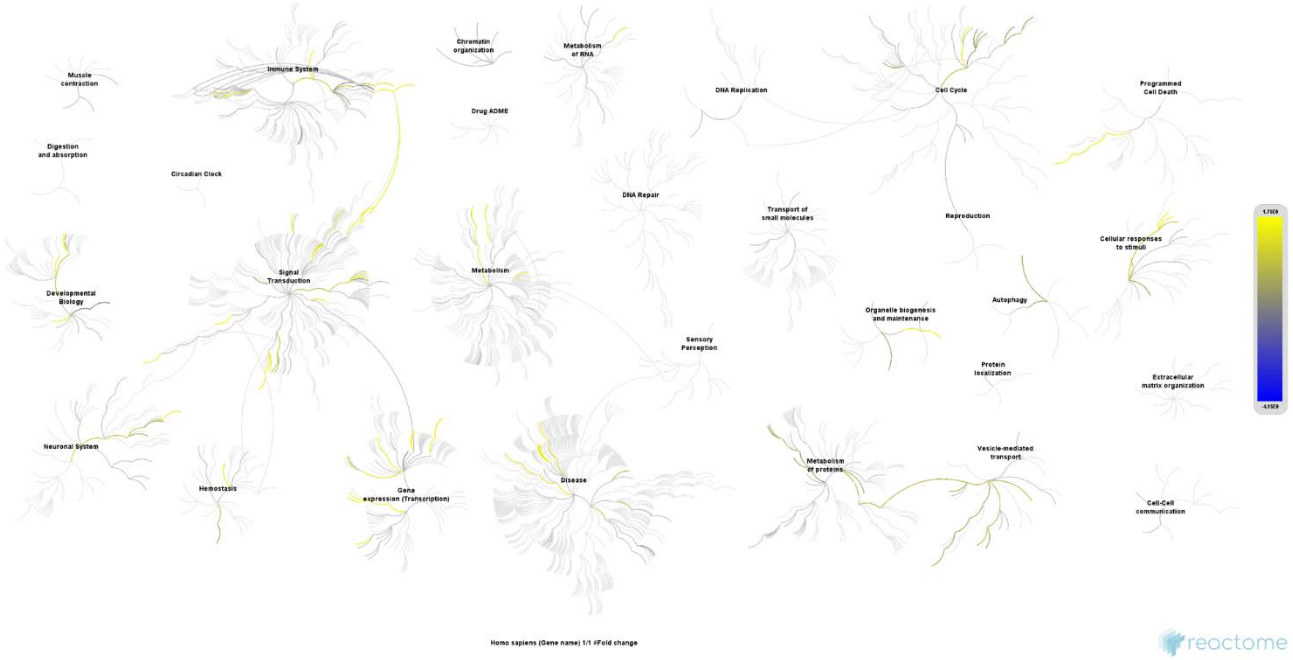

Figure S17: TL most significant proteins displayed in a Reactome genome-wide, hierarchical visualization of pathways in a space filling graph relating to Figure 1B. Highest up and down regulated pathways are depicted in yellow and blue.

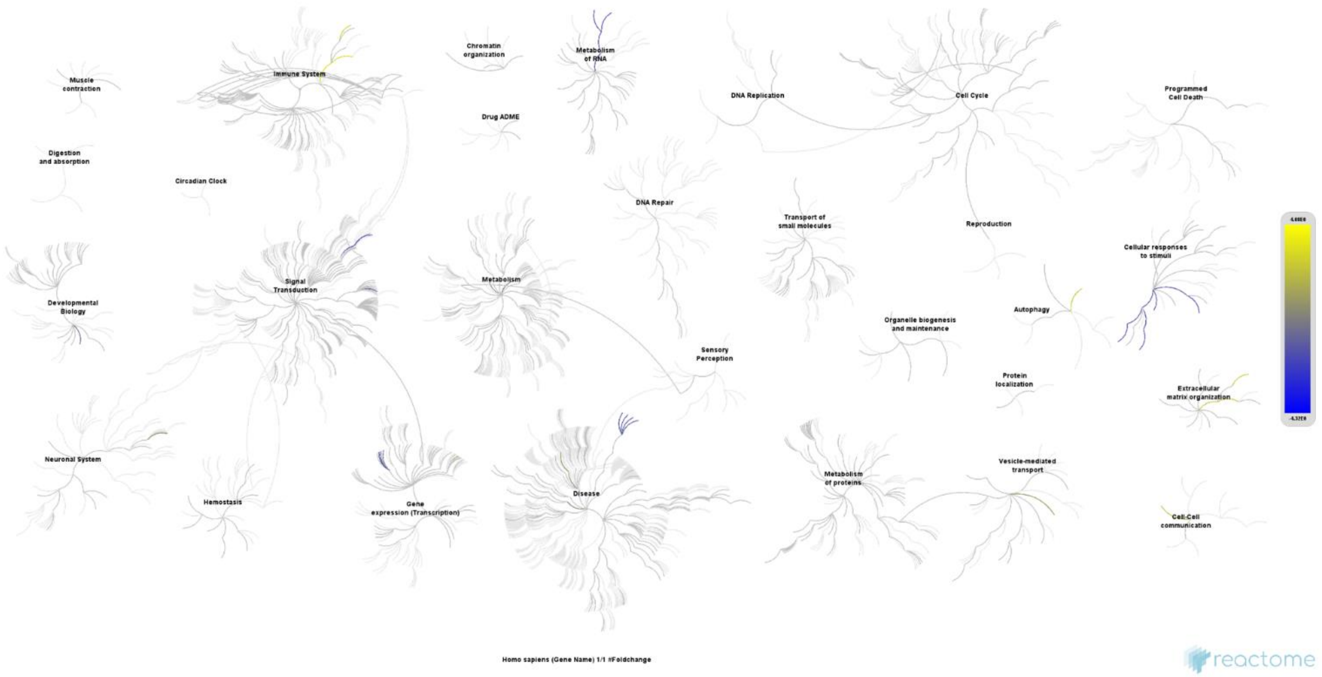

Figure S18: Transcriptomics outcome for the most significant genes displayed in a Reactome genome -wide, hierarchical visualization of pathways in a space filling graph relating to STAR methods qualification and quantification of mRNA. Highest up and down regulated pathways are depicted in yellow and blue.

Supplemental Tables

Pathway Name	Stable Identifier
Prefoldin mediated transfer of substrate to CCT/TriC	(R-HSA-389957)
Cooperation of Prefoldin and TriC/CCT in actin and tubulin folding	(R-HSA- 389958))
Respiratory electron transport, ATP synthesis by chemiosmotic coupling, and heat production by uncoupling proteins.	(R-HSA-163200)
Respiratory electron transport	(R-HSA-611105)
Gene and protein expression by JAK-STAT signaling after Interleukin-12 stimulation	(R-HSA-8950505)
Interleukin-12 signaling	(R-HSA-9020591)
The citric acid (TCA) cycle and respiratory electron transport	(R-HSA-1428517)
Chaperonin-mediated protein folding	(R-HSA-390466)
Interleukin-12 family signaling	(R-HSA-447115)
Protein folding	(R-HSA-391251)
Complex I biogenesis	(R-HSA-6799198)
Beta oxidation of octanoyl-CoA to hexanoyl-CoA	(R-HSA-77348)
Beta oxidation of lauroyl-CoA to decanoyl-CoA-CoA	(R-HSA-77310)
Beta oxidation of hexanoyl-CoA to butanoyl-CoA	(R-HSA-77350)
Mitochondrial protein import	(R-HSA-1268020)
Beta oxidation of decanoyl-CoA to octanoyl-CoA-CoA	(R-HSA-77346)
Depolymerisation of the Nuclear Lamina	(R-HSA-4419969)
RHOBTB2 GTPase cycle	(R-HSA-9013418)
Initiation of Nuclear Envelope (NE) Reformation	(R-HSA-2995383)
Protein localization	(R-HSA-9609507)
RHOBTB GTPase Cycle	(R-HSA-9706574)
Breakdown of the nuclear lamina	(R-HSA-352238)
Drug resistance in ERBB2 KD mutants	(R-HSA-9665230)
Drug-mediated inhibition of ERBB2 signaling	(R-HSA-9652282)
Resistance of ERBB2 KD mutants to AEE788	(R-HSA-9665250)

Table S2.2: **The list of most significantly regulated Reactome pathways identified from the EL proteomics entity input relating to Figure 1A.**

Pathway Name	Stable Identifier
Interleukin-12 family signaling	(R-HSA-447115)
COPI-mediated anterograde transport	(R-HSA-6807878)
L1CAM interactions	(R-HSA-373760)
Interleukin-12 signaling	(R-HSA-9020591)
Senescence-Associated Secretory Phenotype (SASP)	(R-HSA-2559582)
Post NMDA receptor activation events	(R-HSA-438064)
Microtubule-dependent trafficking of connexons from Golgi to the plasma membrane	(R-HSA-190840)
Recycling pathway of L1	(R-HSA-437239)
Transport of connexons to the plasma membrane	(R-HSA-190872)
Signaling by Interleukins	(R-HSA-449147)
ER to Golgi Anterograde Transport	(R-HSA-199977)
Post-chaperonin tubulin folding pathway	(R-HSA-389977)
Activation of NMDA receptors and postsynaptic events	(R-HSA-442755)
Signaling by MAP2K mutants	(R-HSA-9652169)
Signaling by ALK fusions and activated point mutants	(R-HSA-9725370)
Signaling by ALK in cancer	(R-HSA-9700206)
RAF-independent MAPK1/3 activation	(R-HSA-112409)
Prefoldin mediated transfer of substrate to CCT/TriC	(R-HSA-389957)
Formation of tubulin folding intermediates by CCT/TriC	(R-HSA-389960)
Gene and protein expression by JAK-STAT signaling after Interleukin-12 stimulation	(R-HSA-8950505)
Negative feedback regulation of MAPK pathway	(R-HSA-5674499)
Cellular Senescence	(R-HSA-2559583)
Activation of AMPK downstream of NMDARs	(R-HSA-9619483)
Formation of the cornified envelope	(R-HSA-6809371)
RHO GTPases activate IQGAPs	(R-HSA-5626467)

Table S2.3: **The list of most significantly regulated Reactome pathways identified from the TL proteomics entity input relating to Figure 1B**.

Pathway Name	Stable Identifier
rRNA processing in the nucleus and cytosol	(R-HSA-8868773)
Major pathway of rRNA processing in the nucleolus and cytosol	(R-HSA-6791226)
rRNA processing	(R-HSA-72312)
Interferon Signaling	(R-HSA-913531)
Transcriptional regulation of white adipocyte differentiation	(R-HSA-381340)
PERK regulates gene expression	(R-HSA-381042)
Loss of MECP2 binding ability to 5hmC-DNA	(R-HSA-9022534)
Response of EIF2AK1 (HRI) to heme deficiency	(R-HSA-9648895)
Response of EIF2AK4 (GCN2) to amino acid deficiency	(R-HSA-9633012)
Translocation of SLC2A4 (GLUT4) to the plasma membrane	(R-HSA-1445148)
ATF4 activates genes in response to endoplasmic reticulum stress	(R-HSA-380994)
Assembly of collagen fibrils and other multimeric structures	(R-HSA-2022090)
Cellular response to starvation	(R-HSA-9711097)
Regulation of MECP2 expression and activity	(R-HSA-9022692)
NGF-stimulated transcription	(R-HSA-9031628)
Loss of MECP2 binding ability to 5mC-DNA	(R-HSA-9022538)
Collagen chain trimerization	(R-HSA-8948216)
ATF6 (ATF6-alpha) activates chaperone genes	(R-HSA-381183)
MECP2 regulates transcription of genes involved in GABA signaling	(R-HSA-9022927)
Reversible hydration of carbon dioxide	(R-HSA-1475029)
ATF6 (ATF6-alpha) activates chaperones	(R-HSA-381033)
Unfolded Protein Response (UPR)	(R-HSA-381119)
Interferon gamma signaling	(R-HSA-877300)

Table S2.4: The list of most significantly regulated Reactome pathways identified from the transcriptomics analysis relating to STAR methods, qualification and quantification of mRNA.

Supplemental Table Legends:

Table S1.1: The complete list of the most significantly regulated genes of the LA of the AF goat model obtained from Transcriptomics analysis **related to STAR methods**, qualification and quantification of mRNA.

Table S1.2: The complete list of the most significantly regulated proteins of the TL fraction from Proteomics analysis, **related to Figure 1B.**

Table S2.1: Integrated Analysis of Proteomics and Transcriptomics **related to STAR methods**. The complete list of pathways obtained from integrated omics analysis of EL, TL proteomics and transcriptomics.

Table S3.1: The complete list of the most significantly regulated proteins identified uniquely in EL fraction **related to Figure 1A**.
